# Natural polymorphisms in the bovine leukemia virus microRNA cluster modulate miRNA expression and host regulatory pathways

**DOI:** 10.1186/s13567-026-01776-0

**Published:** 2026-05-21

**Authors:** Aneta Pluta, Kerstin Skovgaard, Konrad Józef Dębski, Jean Marie Peloponese, Madison Sokacz, Celima Mourouvin, Tasia Marie Taxis

**Affiliations:** 1 Department of Virology and Viral Animal Diseases, National Veterinary Research Institute, 24-100 Puławy, Poland; 2 Department of Research Support, National Veterinary Research Institute, 24-100 Puławy, Poland; 3 Department of Biotechnology and Biomedicine, Technical University of Denmark, 2800 Kgs. Lyngby, Denmark; 4 Fork Systems, 05-850 Duchnice, Poland; 5 Université Montpellier (UM), 34000 Montpellier, France; 6 Institut de Recherche en Infectiologie de Montpellier (IRIM), CNRS, 34293 Montpellier, France; 7 Department of Animal Science, College of Agriculture and Natural Resources, Michigan State University, East Lansing, MI 48824 USA

**Keywords:** BLV, viral microRNA, single-nucleotide polymorphism, Pol III promoter, innate immunity, tumor suppressor pathways, viral persistence

## Abstract

Bovine leukemia virus (BLV) encodes a cluster of viral microRNAs (miRNAs) that remain abundantly expressed during latency, when viral protein production is restricted. Naturally occurring single-nucleotide polymorphisms (SNPs) within this locus are common, yet their functional consequences for miRNA output and host gene regulation remain poorly defined. Peripheral blood leukocytes from 53 naturally BLV-infected cattle were analyzed, and the 554-nt BLV miRNA locus was amplified and sequenced. Field-derived variants containing mutations within RNA polymerase III promoter motifs, seed regions, and termination signals were selected for functional evaluation. Reference and variant loci were co-expressed with a BLVΔ-miRNA infectious clone in HEK293T cells under controlled conditions. Mature miRNA levels were quantified by stem-loop reverse-transcription quantitative polymerase chain reaction (RT–qPCR), and global host transcriptional responses were assessed using oligonucleotide microarrays. Predicted miRNA targets were identified using bioinformatic analyses. Sequence analysis identified 84 polymorphic sites, with a substantial proportion mapping to Pol III regulatory elements and seed regions. Variant loci displayed altered accumulation of selected mature miRNAs and shifts in predicted seed-dependent target repertoires. Transcriptome profiling revealed variant-associated modulation of innate immune-signaling components, interferon-responsive genes, antigen-presentation pathways, tumor-suppressor networks, and extracellular matrix-related processes. Enrichment analysis demonstrated a statistically significant overlap between predicted miRNA targets and downregulated transcripts. Natural polymorphisms within the BLV miRNA cluster modulate miRNA expression and are associated with distinct host regulatory signatures in a controlled experimental system. These findings suggest that sequence variation in the viral miRNA locus may contribute to differential host–virus interactions and influence mechanisms supporting viral persistence.

**Supplementary Information:**

The online version contains supplementary material available at 10.1186/s13567-026-01776-0.

## Introduction

Bovine leukemia virus (BLV) is a deltaretrovirus that infects bovine B lymphocytes and establishes lifelong persistence [1]. Following proviral integration, BLV deploys a multifaceted regulatory network comprising viral proteins, virus-encoded microRNAs (miRNAs), and long noncoding RNAs (lncRNAs) to reprogram host pathways and promote latency [2]. The essential role of the Tax protein in replication and oncogenic transformation is well established [1, 3], and accumulating evidence indicates that BLV-encoded miRNAs further fine-tune host gene expression and contribute to viral persistence [4, 5].

MicroRNAs are small noncoding RNAs that post-transcriptionally regulate gene expression by binding complementary sequences in target messenger RNAs (mRNAs), leading to translational repression or mRNA decay [6]. miRNA-mediated regulation is documented across distinct retroviral lineages within the Retroviridae family. For example, the gammaretrovirus avian leukosis virus subgroup J (ALV-J) and simian foamy virus (SFV; subfamily Spumaretrovirinae) encode viral miRNAs that alter host gene expression to enhance replication, pathogenesis, and persistence. ALV-J miRNAs remodel proliferative signaling networks [7], whereas SFV miRNAs mimic host miRNAs involved in immune regulation and cell proliferation [5, 8–10]. These observations indicate that miRNA-mediated host reprogramming represents a conserved strategy across divergent retroviral genera.

In BLV, five independent RNA polymerase III (Pol III) transcription units each generate a single hairpin precursor (pre-miRNA) that is subsequently processed into 5p and 3p mature strands [2, 4, 5]. Each compact unit contains type 2 internal Pol III promoter elements—an A-box-like motif adjacent to the 5′ end of the hairpin and a B-box-like motif downstream of the pre-miRNA, followed by a short homopolymeric thymidine tract serving as the terminator [11]. The unusual placement of the Pol III terminator between the A- and B-box elements demonstrates that each pre-miRNA functions as an independent transcriptional unit [11, 12]. Recent analyses further revealed a degenerate array of overlapping A-box-like motifs, including cryptic and triply overlapping sites, as well as tandem B-box-like elements, suggesting that BLV deploys a more complex and unconventional Pol III regulatory architecture [13].

BLV-encoded miRNAs are thought to rewire host signaling networks, conferring a survival advantage that facilitates immune evasion without requiring robust viral protein expression [13, 14]. Genetic ablation of the miRNA cluster markedly attenuates viral replication, underscoring its essential role in BLV persistence and pathogenicity [15]. Despite this recognized importance, the molecular consequences of naturally occurring single-nucleotide polymorphisms (SNPs) within the BLV miRNA locus remain poorly defined. In particular, it is unclear whether sequence variability affects miRNA biogenesis, stability, or target specificity in ways that alter host regulatory networks.

Evidence from other viruses encoding their own miRNAs suggests that such variation may be functionally significant. In gammaherpesviruses such as Epstein–Barr virus (EBV) and Kaposi’s sarcoma-associated herpesvirus (KSHV), SNPs within viral miRNAs can reconfigure latency programs, enhance immune evasion, and modulate pathogenic potential [16]. Similar observations have been reported in murine cytomegalovirus (MCMV), where miRNA sequence variation influences NK- and CD4^+^ T cell responses and persistent infection, and in hepatitis C virus (HCV), where mutations in miRNA-interacting regions alter replication dynamics under interferon pressure [17]. Together, these studies support the concept that sequence polymorphisms within viral miRNA loci may represent an adaptive layer of regulatory diversification.

Building on these observations, the present study investigates whether naturally occurring SNPs within BLV miRNA-coding sequences influence miRNA biogenesis and functional output, and whether such variation reshapes miRNA–mRNA target interactions in ways that modulate cellular pathways involved in antiviral immunity, viral persistence, and early oncogenic processes.

## Materials and methods

### Sample collection

A total of 53 whole-blood samples from cattle naturally infected with BLV were collected through local diagnostic laboratories in Poland within the enzootic bovine leukosis (EBL) surveillance program and transferred to the National Veterinary Research Institute for analysis (Additional file 1). Peripheral blood leukocytes (PBLs) were isolated by centrifugation at 1500 *g* for 25 min, followed by erythrocyte lysis by osmotic shock (H_2_O and 4.5% NaCl), as previously described [18]. Genomic DNA was extracted using the DNeasy Blood and Tissue Kit (Qiagen, Valencia, USA) and stored at −20 °C.

### Polymerase chain reaction (PCR) amplification and sequencing of the miRNA-coding region

The BLV miRNA-coding region was amplified by nested PCR using previously described primers [19] and PrimeSTAR GXL DNA Kit (Takara Bio, Kyoto, Japan). First-round PCR consisted of an initial denaturation at 98 °C for 2 min, followed by 35 cycles of 98 °C for 15 s, 55 °C for 25 s, and 68 °C for 3 min, and then a final extension at 72 °C for 10 min. The second PCR used identical cycling, except for an annealing temperature of 70 °C and a 1-min extension at 68 °C. Amplicons were sequenced by Sanger sequencing using 3730xl DNA Analyzer with BigDye Terminator version 3.1 (Applied Biosystems, Waltham, MA, USA).

### Sequence analysis

Raw sequencing reads were assembled in Geneious Prime 2025.1.2 (Biomatters Ltd., Auckland, New Zealand) to generate consensus sequences, which were deposited in GenBank under accession nos. PV185290–PV185338 and MW470848–MW470851. Alignments were performed using the ClustalW algorithm. RNA polymerase III A-box-like and B-box-like promoter motifs were mapped according to Pluta et al. [13]. miRNA seed regions and transcription termination signals were annotated following Kincaid et al. [4, 5, 11]. Secondary structures of mature miRNAs were predicted using RNAfold web server [20]. Structural accessibility changes relative to the reference sequence were expressed as ΔP_unfolded. Differences between predicted secondary structures were quantified using Hamming distance.

### Cloning of the miRNA-coding locus

Selected BLV miRNA loci were amplified using PrimeSTAR GXL DNA polymerase (Takara Bio, Kyoto, Japan). Amplicons were incubated with Taq DNA polymerase at 72 °C for 30 min to generate 3′-A overhangs, purified, and ligated into the promoterless pDrive vector using the Qiagen PCR Cloning Kit (Qiagen, Hilden, Germany). A reference miRNA-coding fragment derived from BLV strain 344 (JC613347.1), belonging to genotype G4 and representative of the predominant genotype among the Polish field isolates analyzed in this study, was cloned using the same protocol [21]. This construct served as the reference locus for functional comparison of naturally occurring variants. The resulting plasmids included pDrive_miRNA/V03, V09, V18, V29, V90, V96, and V99 and the reference construct pDrive_miRNA/344. The empty control vector was generated by EcoRI digestion of pDrive followed by self-ligation with T4 DNA ligase (Thermo Fisher Scientific, Waltham, MA, USA).

### Cell culture, transfection, and RNA isolation

HEK293T cells were cultured in Dulbecco’s modified Eagle medium (DMEM; Lonza BioWhittaker, Verviers, Belgium) supplemented with 4.5 g/L glucose, 2 mM l-glutamine, 10% heat-inactivated fetal bovine serum (Gibco, Thermo Fisher Scientific, USA), and 1% penicillin–streptomycin (Lonza BioWhittaker) at 37 °C in 5% CO_2_. Cells were seeded at 2 × 10^5^ per well in six-well plates (Nunc, Thermo Fisher Scientific) 24 h prior to transfection. On the day of transfection, cells were cotransfected with pDrive_miRNA constructs and the miRNA-deficient BLV infectious clone pBLVΔ-miRNA. Plasmid DNA was diluted in serum-free medium and complexed with TransIT-LT1 (Mirus Corporation, Madison, WI, USA) at 2 µL of reagent per 1 µg of DNA for 15 min at room temperature. Experimental groups comprised seven field-derived variants and one reference construct. Control conditions included pDriveΔmiRNA alone and pDriveΔmiRNA cotransfected with pBLVΔ-miRNA. Then, 48 h after transfection, cells were harvested by scraping and pelleted by centrifugation. Total RNA was isolated using the NucleoSpin RNA Plus Kit (Macherey–Nagel, Düren, Germany). Only samples with RNA integrity number ≥ 8.5 were used for downstream analyses. All conditions were performed in biological triplicate (Table 1).

**Table 1:** **Transfection setup for BLV miRNA variants and reference and control groups**

Group type	Variant(s)	Plasmid(s)
Experimental	V03, V09, V18, V29, V90, V96, V99	pDrive_miRNA/variant + pBLVΔ-miRNA
Reference	344 (wild type)	pDrive_miRNA/344 + pBLVΔ-miRNA
Control 1	–	pDrive∆miRNA
Control 2	–	pDrive∆miRNA + pBLVΔ-miRNA

Seven experimental groups received field-derived BLV miRNA variants cotransfected with pBLVΔ-miRNA; the reference group carried wild-type miRNA (344); controls included pDrive∆miRNA alone or cotransfected with pBLVΔ-miRNA.

### Quantification of miRNA

Complementary DNA (cDNA) was synthesized from 100 ng of total RNA, following the method described by Balcells et al. [22]. Each 10-µL reaction contained 10× poly(A) polymerase buffer, ATP at 0.1 mM, RT primer at 1 µM with the sequence 5′-CAGGTCCAGTTTTTTTTTTTTVN, dNTP at 0.1 mM each, 100 U MuLV reverse transcriptase, and 1 U poly(A) polymerase (New England Biolabs, Ipswich, MA, USA). Reactions were incubated at 42 °C for 60 min and then heat-inactivated at 95 °C for 5 min. Primers for BLV miRNAs, blv-miR-B1 to blv-miR-B5, and for human miRNAs, hsa-miR-34a and hsa-miR-20a, were designed using miRprimer [23]. Primer sequences are provided in Additional file 2. Quantitative PCR was performed in 20-µL reactions using QuantiTect SYBR Green Master Mix (Qiagen, Hilden, Germany) on a Rotor-Gene Q (Qiagen). hsa-miR-20a was used as the endogenous reference, and the reference construct BLV 344 served as the calibrator. Relative expression was calculated using the ΔΔ*C*_t_ method [22], where Δ*C*_t_ = *C*_t_ (target) − *C*_t_ (hsa-miR-20a), ΔΔ*C*_t_ = Δ*C*_t_ (variant) − Δ*C*_t_ (calibrator), and fold change = 2^−ΔΔCt^.

### Quantification of viral mRNAs

Total RNA isolated from transfected HEK293T cells was treated with DNase I to eliminate potential residual DNA. Reverse transcription was performed using the NG dART RT kit at 47 °C for 50 min. The resulting cDNA served as template to quantify BLV gag, env, and tax/rex transcripts. qPCR reactions were performed using 2× QuantiTect SYBR Green Master Mix (Qiagen, Hilden, Germany) and gene-specific primers (Additional file 3) on a Rotor-Gene Q (Qiagen). Viral transcript levels were normalized to HPRT expression. Relative expression was calculated using the ΔΔ*C*_t_ method [24]. Fold-change values were determined relative to control 1 for each miRNA variant and for reference construct.

### Microarray-based transcriptional profiling of transfected HEK293T cells

Transcriptome profiling was performed for V29 and V90 variants and the reference and control 2. These groups were selected for detailed comparative analysis on the basis of their distinct miRNA expression patterns. For each condition, equal amounts of RNA obtained from three independent transfections were pooled to generate representative samples. Pooled RNA was analyzed in six technical replicates using the Agilent Two-Color Low Input Quick Amp Labeling Kit (Agilent Technologies, Santa Clara, CA, USA). Briefly, 100 ng of total RNA was reverse-transcribed and amplified into cDNA, followed by fluorescent labeling with Cy3 (control) or Cy5 (experimental). Labeled cRNA was purified and quantified using a Nanophotometer Pearl (IMPLEN, Munich, Germany). For each hybridization, 300 ng of Cy5-labeled pooled cRNA from V29, V90, reference, or control 2 was combined with 300 ng of Cy3-labeled pooled cRNA from control 1 (Table 1). Hybridization was performed on Agilent 072363 SurePrint G3 Human GE v3 8 ×  60 K microarrays, containing 26,083 unique Entrez genes and 30,606 unique lncRNAs, in accordance with the manufacturer’s protocol. Arrays were scanned using an Agilent G2505C scanner. Raw image data were processed with Agilent Feature Extraction version 12.0.3.1, including background correction, outlier removal, and dye-bias normalization. The complete dataset has been deposited in the NCBI Gene Expression Omnibus under accession no. GSE299212.

### Microarray data analysis and pathway enrichment

Processed microarray data were analyzed using GeneSpring GX 14.8 (Agilent Technologies). Default two-color normalization procedures were applied and quality control metrics were evaluated prior to downstream analysis. Differentially expressed genes were defined using a fold-change threshold of |FC| ≥ 1.5 combined with one-way analysis of variance (ANOVA) across groups and Benjamini–Hochberg correction for multiple testing. Genes with false discovery rate (FDR) < 0.05 were considered significant. To interpret biological relevance, lists of differentially expressed genes (DEGs) were subjected to canonical pathway enrichment analysis using Ingenuity Pathway Analysis (IPA) (Qiagen) with Fisher’s exact test against the Ingenuity Knowledge Base reference dataset [25].

### qPCR-based reference gene selection and validation of differentially expressed genes

High-throughput qPCR was performed using Dynamic Array IFC 48.48 chips on the BioMark HD system (Fluidigm) with TaqMan Gene Expression Master Mix (Life Technologies) and EvaGreen (VWR Bie & Berntsen) detection chemistry. This platform was applied to identify a stable endogenous reference gene in HEK293T cells from a panel comprising ATP5F1, PGK1, HPRT1, GAPDH, ACTB, RPL13A, RPLP0, YWHAZ, DDX5, and CAPZB. In parallel, expression of genes previously associated with BLV miRNA regulation was examined, including FOS, MAP2K1, PPT1, TPT1, CDKN1A, TLE2, STAT3, CHD3, CYSLTR1, DST, KLF11, NCF2, EPAS1, CDCA7, HBP1, ELL2, TNFAIP6, IL1A, CXCL2, IRF1, PIAS4, HSF2, ANXA1, CREM, CXCL8, and EGR1 [4]. For independent validation of microarray results, eight DEGs (ATP7A, CHD6, CXCL1, EGR1, KLF12, RPL23AP32, RPL37A, and TNFAIP6) were selected. Primer sequences are provided in Additional file 4. Relative expression values were normalized to HPRT1.

### Target prediction and sequence retrieval

Putative targets of BLV-encoded miRNAs were predicted independently in human and bovine transcriptomes using miRanda version 3.3a following the algorithm described by Enright et al. [26]. Target prediction was restricted to predefined sets of differentially expressed genes identified in the microarray analysis. For each gene, both 3′ untranslated regions (3′UTR) and coding sequences (CDS) were retrieved from Ensembl release 113 using the BiomaRt Python package (version 0.8.0). Sequences of lncRNAs not available in Ensembl were obtained from LNCipedia version 2.1 and version 5.2. Predicted miRNA–mRNA interactions were filtered using a minimum alignment score ≥ 140 and binding free energy ΔG ≤  −20 kcal/mol. Orthology relationships between Homo sapiens and Bos taurus genes were retrieved from Ensembl release 113. Functional enrichment analyses were performed using g:Profiler (g:GOSt) with Benjamini–Hochberg correction at *α* = 0.05, querying Gene Ontology (GO) categories, Human Phenotype Ontology, Human Protein Atlas annotations, miRNA-related terms, and WikiPathways. Where applicable, bovine orthologs were mapped to human gene identifiers to facilitate cross-species comparison.

### Statistical analysis

Expression levels of BLV miRNA variants and viral transcripts (*tax*/*rex*, *gag*, *env*) were compared using the nonparametric Kruskal–Wallis test implemented in Statistica 10. A *p* < 0.05 was considered statistically significant. Enrichment of predicted miRNA targets among DEGs was evaluated using the hypergeometric distribution and Fisher’s exact test implemented in R. The expected random overlap was calculated as *E* = *n* × (*K*/*N*), where *N* represents the total number of genes on the array, *K* the number of predicted targets, *n* the number of DEGs, and *k* the observed overlap. Fold enrichment was calculated as *k*/*E*, and effect size was expressed as the odds ratio derived from a 2 × 2 contingency table. One-sided and two-sided *p*-values were reported as appropriate.

## Results

### SNP identification in the miRNA-coding locus

**Figure 1:**
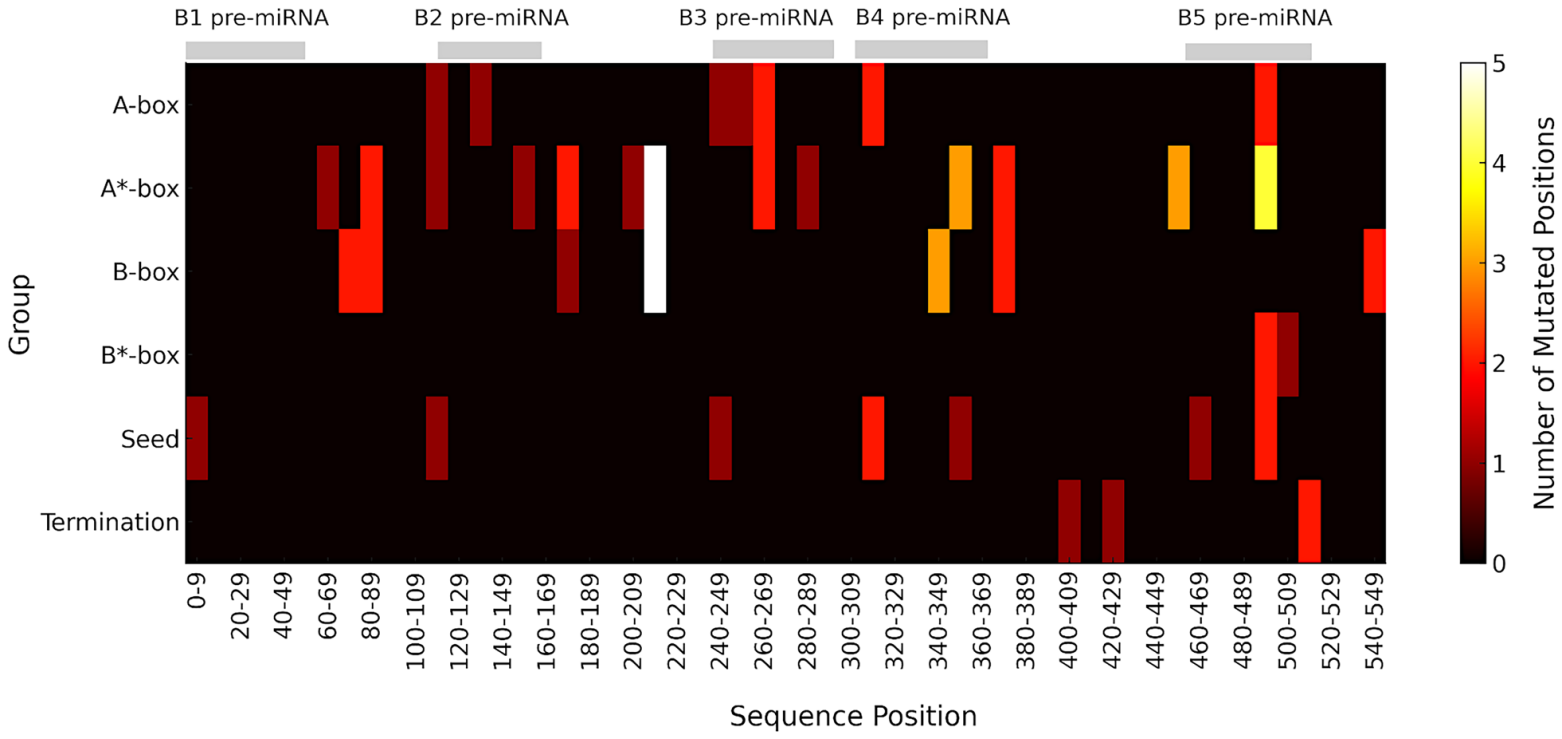
**Distribution and density of SNPs within BLV regulatory motifs.** Heatmap displaying SNP density across sequence motifs. The *x*-axis represents genomic intervals from position 0 to 554, grouped into 10-nt bins. The *y*-axis lists motif categories: A-box-like, cryptic A*-box-like, B-box-like, cryptic B*-box-like, seed, and termination signal. The color scale indicates the number of SNP positions per bin. A value of 0 (dark) indicates no SNPs, while higher values indicate the number of distinct SNP positions in the bin. Locations of pre-miRNAs B1–B5 are marked above the heatmap in gray.

To assess nucleotide variability within the BLV miRNA locus, 53 sequences, each 554 nucleotides (nt) long, were compared with the JC613347 reference sequence (BLV 344) (Additional file 5). The alignment revealed 84 polymorphic sites, including 80 single-nucleotide polymorphisms (SNPs) and 4 insertion/deletion (InDel) events. Variants were mapped to specific regulatory elements (Additional file 6). Of the 84 polymorphisms, 61 (72.6%) localized within regulatory regions. The cryptic A*-box harbored the highest number of SNPs (*n* = 28), followed by the B-box (*n* = 19), with lower counts in the A-box (*n* = 10), seed regions (*n* = 9), cryptic B*-box (*n* = 3), and the termination signal (*n* = 4) (Figure 1).

### Detailed analysis of SNPs within miRNA functional motifs of selected BLV isolates

Seven BLV isolates harboring 33 mutations within key regulatory motifs were selected for in-depth analysis on the basis of unique patterns of SNPs and InDels across the BLV miRNA locus. The selected isolates carried polymorphisms spanning seed regions, A-box–like, B-box–like, A*-box–like, B*-box–like promoter motifs, and terminator sequences, yielding distinct polymorphic signatures that could underlie differences in miRNA output and regulatory activity (Table 2).

**Table 2:** **Nucleotide polymorphisms in BLV field sequences within the miRNA loci relative to regulatory elements**

Nucleotide position	Localization of SNPs relative to regulatory elements	Reference and variant sequences							
		JC613347	V18	V03	V96	V99	V90	V09	V29
6	w/miR-B1-5p seed; bd. miR-B1 A1-box	G	⸱	⸱	⸱	⸱	⸱	⸱	A
67	w/miR-B1 A*-box; bd. miR-B1 B1-box	A	⸱	⸱	⸱	⸱	⸱	⸱	G
70	w/miR-B1 B1-/A*-box	G	⸱	A	A	A	A	A	A
106	vic. miR-B2 A*-box	G	⸱	⸱	⸱	⸱	A	⸱	⸱
137	w/miR-B2 A3-box	T	⸱	⸱	⸱	⸱	⸱	G	⸱
154	w/miR-B2 A*-box; bd. miR-B2-3p seed	A	⸱	⸱	⸱	⸱	G	⸱	⸱
169	adj. miR-B2 term./A*-box	A	⸱	⸱	⸱	G	⸱	⸱	⸱
170	bd. miR-B2 A*-box; vic. miR-B2 term	T	⸱	⸱	⸱	⸱	C	⸱	⸱
174	w/miR-B2 A*-box; bd. B1-box	C	⸱	T	⸱	⸱	⸱	⸱	⸱
193	adj. miR-B2 B2-box	C	⸱	⸱	⸱	⸱	⸱	⸱	T
210	w/miR-B2 B3-/A*-/A*-box	G	⸱	⸱	⸱	⸱	⸱	⸱	A
211	w/miR-B2 B3-/A*-/A*-box	G	A	A	A	A		A	
212	w/miR-B2 B3-/A*-/A*-box	T	⸱	⸱	⸱	⸱	⸱	⸱	A
247	w/miR-B3-5p seed; w/miR-B3 A1-box	T	⸱	⸱	⸱	C	⸱	⸱	⸱
253	w/miR-B3 A1-box	C	⸱	⸱	T	⸱	⸱	⸱	⸱
267	w/miR-B3 A3-/A*-box	A		T	⸱	⸱	⸱	⸱	⸱
284	w/miR-B3 A*-box; vic. miR-B3-3p seed	G	A	⸱	⸱	⸱	⸱	⸱	⸱
310	w/miR-B4-5p seed; w/miR-B4 A1-/A*-box	A	⸱	⸱	⸱	⸱	⸱	⸱	Del
314	w/miR-B4-5p seed	A	G	⸱	⸱	⸱	⸱	⸱	G
314	w/miR-B4 A1-box	A	G	⸱	⸱	⸱	⸱	⸱	G
341	w/miR-B3 B1-box	C	⸱	⸱	⸱	⸱	⸱	⸱	T
347	bd. miR-B3 B1-box; bd. miR-B4 A*-box; adj. miR-B4-3p seed	C	⸱	⸱	⸱	⸱	⸱	⸱	T
427	w/miR-B4 term.; adj. miR-B4 B2-/A*-box	T	⸱	C	C	C	⸱	C	⸱
448	bd. miR-B5 A*-box	T	⸱	⸱	⸱	⸱	⸱	⸱	A
454	w/miR-B5 A*-box	C	⸱	⸱	⸱	⸱	⸱	⸱	T
456	w/miR-B5 A*-box	C	⸱	⸱	⸱	⸱	⸱	⸱	T
462	adj. miR-B5-5p seed; vic. miR-B5 A1**-/A*-box	–	⸱	⸱	⸱	⸱	⸱	⸱	Ins T
463	bd. miR-B5-5p seed; adj. miR-B5 A1**-box	A	⸱	⸱	⸱	⸱	⸱	⸱	G
490	w/miR-B5 A3-box; bd. miR-B5 A*-box	A	⸱	⸱	⸱	⸱	⸱	⸱	Del
498	w/miR-B5-3p seed; w/miR-B5 A*-/B*-box; adj. miR-B5 A3-box	G	⸱	⸱	⸱	⸱	⸱	⸱	A
499	w/miR-B5-3p seed; w/miR-B5 A*-/B*-box; adj. miR-B5 A3-box	A	⸱	⸱	⸱	⸱	⸱	⸱	G
549	w/miR-B5 B1-/A*-box	A	G	G	G	G	G	G	⸱
551	w/miR-B5 B1-/A*-box	A	⸱	⸱	⸱	⸱	G	⸱	⸱
554	bd. miR-B5 B1-box; adj. miR-B5 A*-box	C	⸱	T	⸱	⸱	⸱	⸱	⸱

The table presents a transposed alignment of seven BLV isolates, V03, V09, V18, V29, V90, V96, and V99, compared with the reference strain JC613347.

Dots indicate nucleotides identical to the reference, while letters (A, T, G, C) represent substitutions. “Del” indicates a deletion, and “Ins T” denotes a thymine insertion. SNPs are annotated according to their position relative to regulatory motifs: within (w/), at the boundary of the regulatory element (bd.), adjacent to the regulatory element (adj.; 1 nt away), or in the vicinity of the regulatory element (vic.; 2–3 nt away).

The isolate V29 showed the broadest accumulation of changes, comprising both SNPs and InDels across multiple key functional motifs. These included substitutions at position 6 within miR-B1-5p seed region and at 463 and 498–499 within miR-B5 seed windows, multiple variants within Pol III promoter motifs (448, 454, 456; 341, 347), two deletions (position 310 within the miR-B4-5p seed/A1–A*-box region and 490 within the miR-B5 A3/A*-box), and a single insertion (position 462, Ins T). This pattern suggests a potentially pronounced impact on miRNA target specificity (seed changes), Pol III promoter function (A/B motifs), and termination efficiency. The remaining isolates harbored predominantly SNPs concentrated at recurrent regulatory hot spots. Frequently shared changes included the B1-/A*-box variant at position 70 (G → A; V03, V96, V99, V90, V09, V29), the B3/A* cluster at position 211 (G → A; V18, V03, V96, V99, V09), and the B1-/A*-box variant at position 549 (A → G; V18, V03, V96, V99, V90, V09). Several isolates also carried a modification within the Pol III termination region at position 427 (T → C; V03, V96, V99, V09).

### Expression analysis of BLV miRNA variants

**Figure 2:**
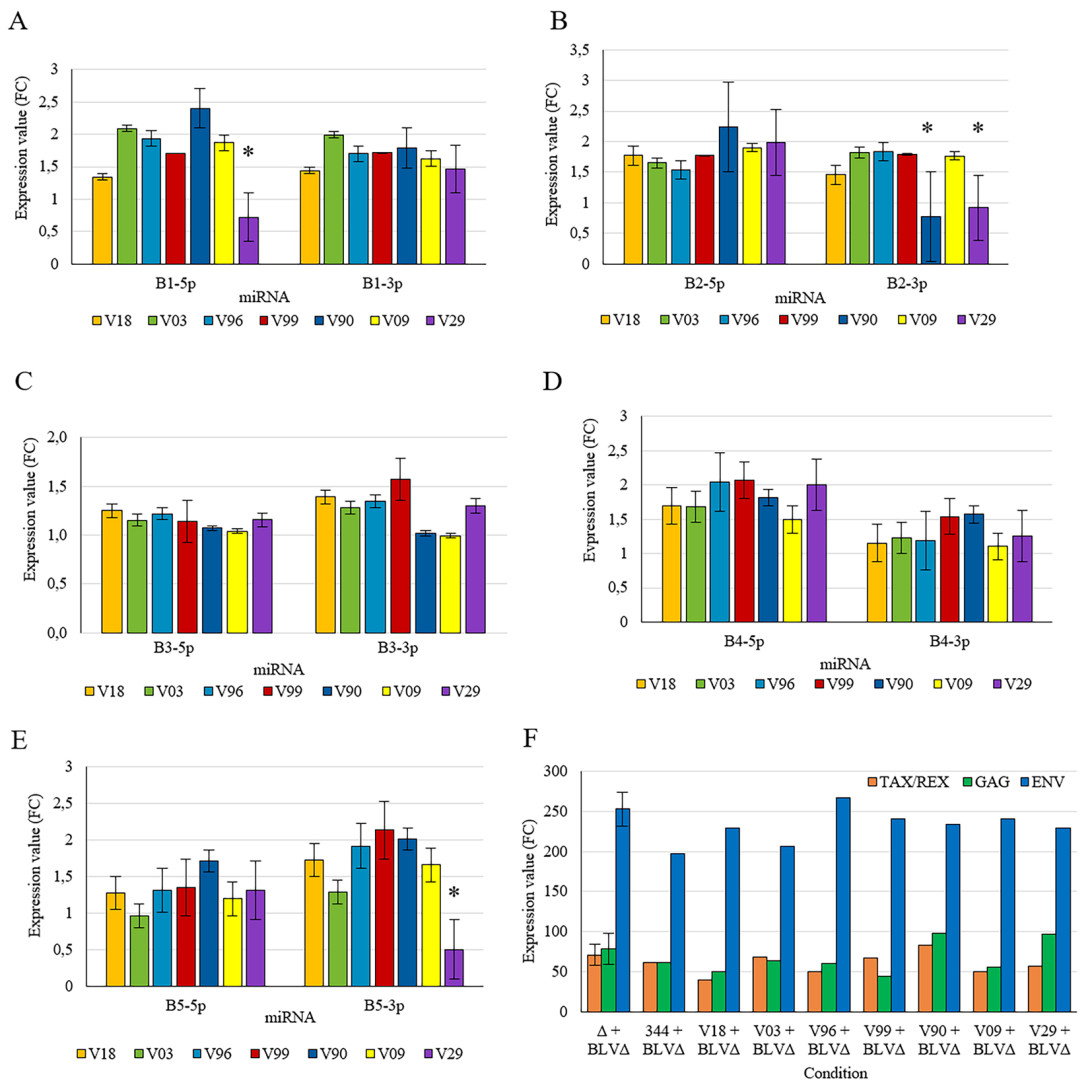
**Expression analysis of BLV miRNA variants and viral genes.** **A**–**E** Relative expression levels of the 5p and 3p strands of BLV-encoded miRNAs measured following transient co-expression of each BLV miRNA locus (reference BLV 344 and variants V03, V09, V18, V29, V90, V96, and V99) together with the BLVΔ-miRNA in HEK293T cells. Expression values were calculated using the 2^−ΔΔCt^ method and are shown as mean fold change ± standard deviation (SD) from independent transfections. Statistical differences between constructs were assessed using the Kruskal–Wallis test (*p* < 0.05). **F** Expression of viral mRNAs encoding Tax/Rex, Gag, and Env under the same transfection conditions (Kruskal–Wallis *p* > 0.43).

To directly assess whether naturally occurring polymorphisms in the BLV miRNA locus affect miRNA production, each of the seven field-derived miRNA loci (V03, V09, V18, V29, V90, V96, and V99) and the reference locus (BLV 344) were cloned and transiently co-expressed with a miRNA-deficient BLV backbone (pBLVΔ-miRNA) in HEK293T cells. The use of the pBLVΔ-miRNA backbone in combination with separate miRNA expression constructs (pDrive_miRNA variants V03, V09, V18, V29, V90, V96, and V99) prevented the generation of functional AS1-L and AS1-S antisense transcripts, which are encoded at the same genomic locus as the miRNAs. In contrast, AS2 originates from a non-overlapping genomic region and was therefore expected to be present at comparable levels across all conditions, thereby minimizing its impact on comparative analyses. HEK293T cells were used as a standardized background because they support highly efficient transfection, robust RNA polymerase III-driven expression of BLV miRNA precursors, and reproducible quantification of mature BLV miRNAs while minimizing variability associated with proviral load, infection stage, and host genetic background. As a result, both 5p and 3p arms were detected for all BLV miRNAs across constructs, with only minor shifts in relative arm usage (Figure 2A–E). Variant V29 showed significantly reduced levels of miR-B1-5p, miR-B2-3p, and miR-B5-3p, and variant V90 showed a significant decrease in miR-B2-3p. In contrast, the remaining variants were comparable to the reference construct, indicating that most polymorphisms do not strongly disrupt miRNA maturation or accumulation. Analysis of viral transcripts (tax/rex, gag, and env) revealed no statistically significant differences among variants (*p* > 0.43; Figure 2F), consistent with comparable transfection efficiency under the experimental conditions.

### Microarray-based analysis of DEGs across experimental groups

#### Expression patterns of coding and noncoding RNAs across experimental conditions

To determine whether polymorphisms within the BLV miRNA locus alter host gene expression, we conducted whole-transcriptome microarray profiling in HEK293T cells transfected with the BLVΔ-miRNA complemented with either the reference miRNA locus (reference), field-derived variant loci (V29 or V90), or an empty add-back control (control 2). Variants V29 and V90 were selected because they showed clear reductions in the accumulation of specific BLV miRNAs, suggesting potentially stronger functional effects on cellular pathways. Microarray analysis, covering 56,689 gene-level entities represented on the array, identified 1747 differentially expressed genes in V29-expressing cells, 1954 in V90-expressing cells, 2034 in control 2-transfected cells, and 2062 in cells expressing the reference locus (Additional file 7). All expression changes were calculated relative to mock-transfected HEK293T cells. The differentially expressed sets were dominated by protein-coding genes and lncRNAs, whereas pseudogenes, snoRNAs, other noncoding RNAs, and uncharacterized loci represented only a minor fraction (3%). In all experimental groups, downregulated genes outnumbered upregulated genes. Across conditions, 621–773 (35.5–39.6%) protein-coding genes and 346–453 (19.8–22.3%) lncRNAs were downregulated (Table 3). The total number of affected genes did not differ substantially among the reference, V29, and V90 constructs. These findings indicate that naturally occurring BLV miRNA variants do not induce a qualitatively distinct transcriptional phenotype but instead influence the magnitude of a shared suppressive program. Thus, BLV miRNAs appear to fine-tune the transcriptional response associated with BLVΔ-miRNA backbone expression and transfection, rather than acting as the sole determinants of observed gene-expression changes.

**Table 3:** **Global distribution of differentially expressed transcripts by biotype in HEK293T cells expressing BLV miRNA loci**

Gene type	Gene expression	Control 2 (%)		Reference (%)		V29 (%)		V90 (%)	
Protein-coding gene	UP	498	(24.5)	480	(23.3)	442	(25.3)	436	(22.3)
	DOWN	678	(33.3)	752	(36.5)	621	(35.5)	773	(39.6)
Pseudogene	UP	10	(0.5)	10	(0.5)	9	(0.5)	9	(0.5)
	DOWN	37	(1.8)	40	(1.9)	33	(1.9)	41	(2.1)
lncRNA	UP	112	(5.5)	127	(6.2)	113	(6.5)	116	(5.9)
	DOWN	453	(22.3)	424	(20.6)	346	(19.8)	380	(19.4)
snoRNA	UP	7	(0.3)	9	(0.4)	8	(0.5)	10	(0.5)
	DOWN	50	(2.5)	49	(2.4)	32	(1.8)	28	(1.4)
Other ncRNAs	UP	5	(0.2)	5	(0.2)	3	(0.2)	7	(0.4)
	DOWN	12	(0.6)	14	(0.7)	7	(0.4)	5	(0.3)
Uncharacterized gene	UP	50	(2.5)	40	(1.9)	35	(2.0)	43	(2.2)
	DOWN	122	(6.0)	112	(5.4)	98	(5.6)	106	(5.4)
Total		2034	(100)	2062	100	1747	100	1954	100

Values indicate the number of significantly upregulated (UP) or downregulated (DOWN) gene-level entities and their percentage of all differentially expressed entities detected under each transfection condition (control 2, reference BLV 344, V29, and V90). In this section, the term “transcript” refers to microarray-represented gene-level entities assigned to biotypes, including lncRNAs.

**Figure 3:**
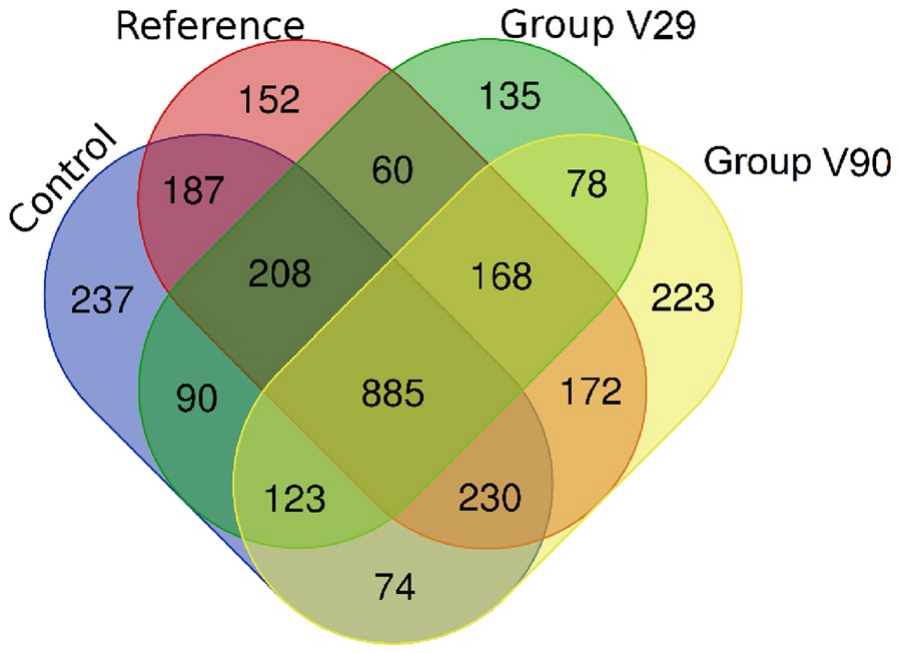
**Differential expression of genes across experimental groups.** Venn diagram illustrating the number of DEGs that are unique to each group, with overlapping regions indicating genes shared between two or more groups.

To further evaluate overlap patterns, a Venn diagram compared differentially expressed genes across control 2, reference, V29, and V90 (Figure 3).

A set of 885 differentially expressed genes was common to all four groups, representing a shared core signature. Pairwise overlaps included 60 genes shared between the reference and V29 groups and 172 genes shared between the reference and V90 groups. Each group also contained a distinct subset of group-specific differentially expressed genes, comprising 152 of 2062 (7.4%) in the reference group, 135 of 1747 (7.7%) in V29, 223 of 1954 (11.4%) in V90, and 237 of 2034 (11.7%) in control 2. Group-specific genes and functional annotations are listed in Additional file 8A–D.

#### Distinct pathway enrichment signatures across experimental groups

**Figure 4:**
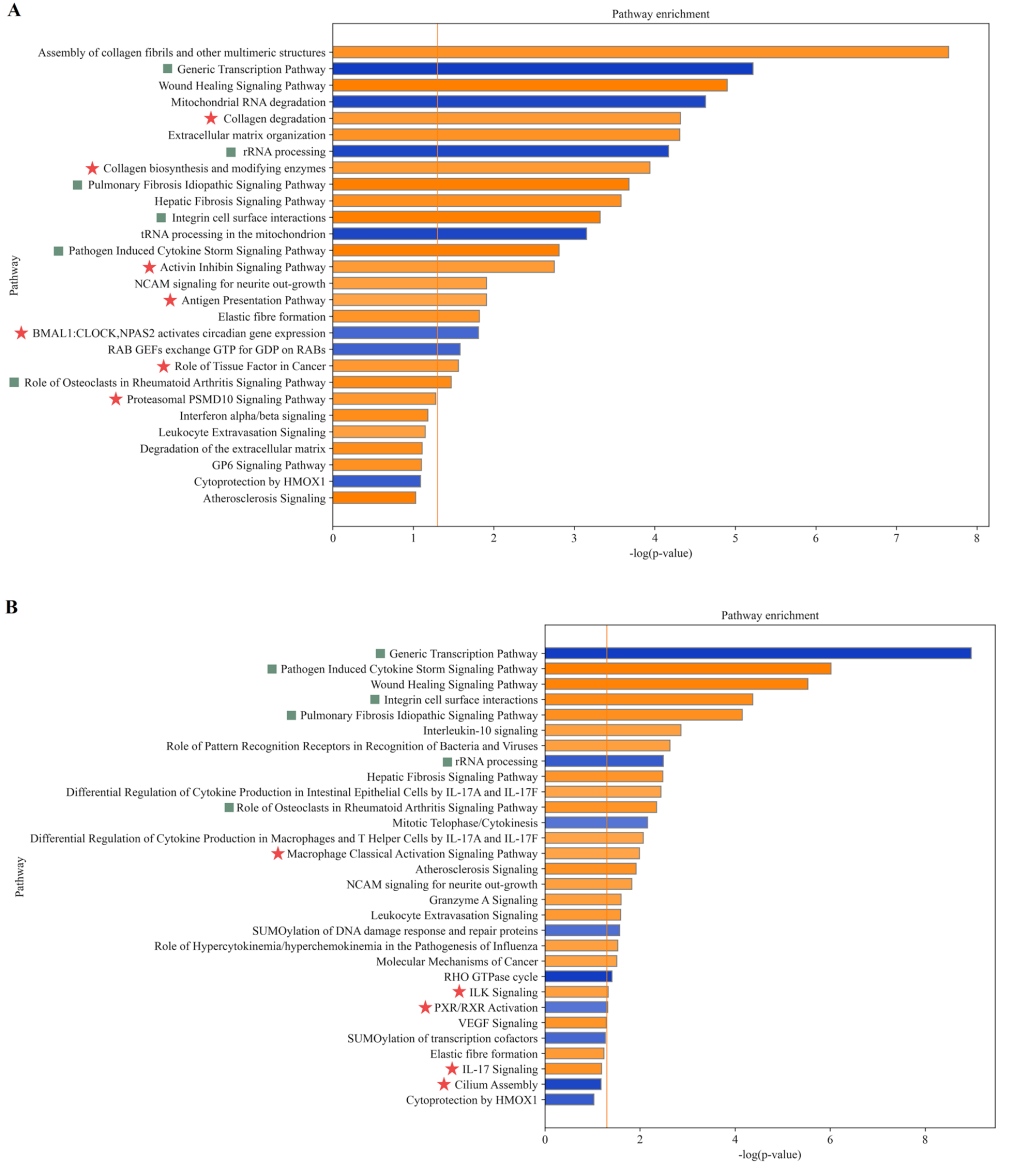
**Pathway enrichment analysis in control 2 (A) and reference (B) groups.** The enrichment significance is shown as −log(*p*-value), reflecting statistical confidence of pathway overrepresentation. A threshold of −log(*p*-value) ≥ 1.3, corresponding to *p* ≤ 0.05, was applied. The *z*-score predicts pathway activation state, with positive values indicating predicted activation and negative values indicating inhibition; only pathways with |*z*-score| > 2.0 are shown. Orange bars represent pathways with positive *z*-score, whereas blue bars correspond to negative *z*-scores. Common pathways are marked with a teal square; group-specific pathways are marked with a red asterisk.

**Figure 5:**
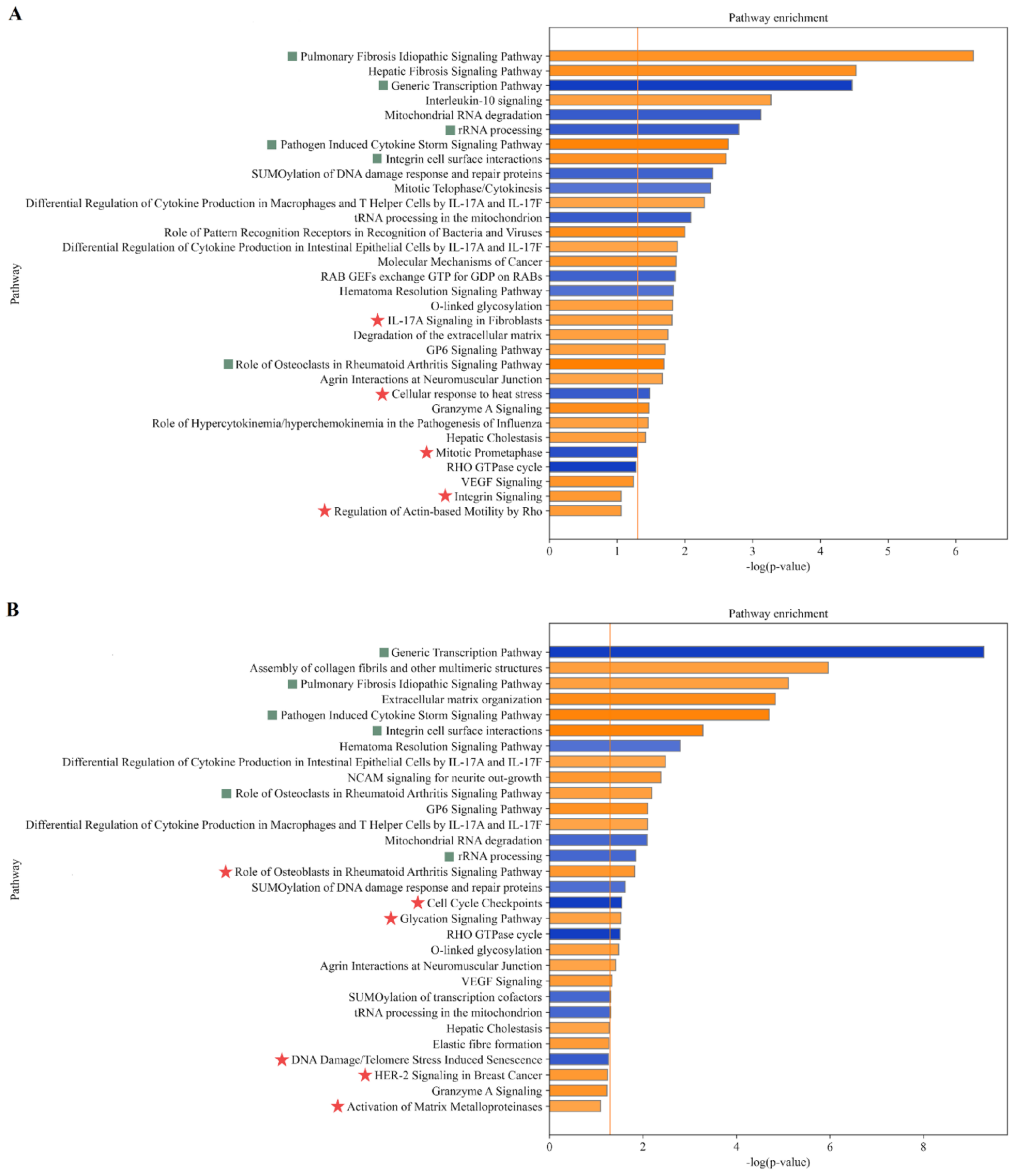
**Pathway enrichment analysis in V29 (A) and V90 (B) groups.** The enrichment significance is shown as −log(*p*-value), reflecting statistical confidence of pathway overrepresentation. A threshold of −log(*p*-value) ≥ 1.3, corresponding to *p* ≤ 0.05, was applied. The *z*-score predicts pathway activation state, with positive values indicating predicted activation and negative values indicating inhibition; only pathways with |*z*-score| > 2.0 are shown. Orange bars represent pathways with positive *z*-score, whereas blue bars correspond to negative *z*-scores. Common pathways are marked with a teal square; group-specific pathways are marked with a red asterisk.

Pathway enrichment analysis identified both shared and group-specific biological processes (Figures 4 and 5). Despite variability in the size of group-specific gene subsets, several canonical pathways were consistently enriched across all groups, including the generic transcription pathway, integrin cell surface interactions, idiopathic pulmonary fibrosis signaling pathway, pathogen-induced cytokine storm signaling pathway, rRNA processing, and the role of osteoclasts in rheumatoid arthritis signaling pathway. These recurring enrichments delineate a common core of transcriptional and signaling programs plausibly attributable to the shared pBLVΔ-miRNA backbone and to cellular responses to plasmid transfection across experimental conditions.

Group-specific enrichments were also observed. In control 2, the profile was dominated by extracellular matrix organization and tissue remodeling, together with immune-related processes and RNA metabolism pathways. Distinctive enrichments included antigen presentation, activin–inhibin signaling, circadian rhythm regulation, and tissue factor- and proteasome-related cancer pathways (Figure 4A). The reference group showed pronounced immune regulation, including enrichment of IL-10 and IL-17 signaling, macrophage activation, pathogen recognition, and hypercytokinemia-related pathways, together with protein metabolism and cell-cycle processes. Distinct pathways included ILK-mediated cell adhesion and growth-factor signaling, metabolic regulation via PXR/RXR activation, and inflammatory and cilium-assembly pathways (Figure 4B). V29 showed enrichment of cell-cycle regulation, RNA metabolism, and cellular stress responses, together with cytokine signaling, cytoskeletal remodeling, vesicle trafficking, and broader metabolic and signaling pathways. Distinctive pathways included IL-17A-mediated fibroblast signaling, cellular stress responses, integrin and cytoskeletal regulation, and mitotic cell-cycle modules (Figure 5A). V90 displayed the broadest enrichment, encompassing extracellular matrix (ECM) remodeling; metabolic, immune and cancer signaling; cell-cycle and transcriptional control; and cellular stress responses, together with glycation- and telomere-associated senescence. Unique pathways included matrix metalloproteinase activation, cell-cycle checkpoint control, DNA damage- and telomere stress-induced senescence, glycation signaling, HER-2-associated oncogenic signaling, and osteoblast-mediated regulation in rheumatoid arthritis (Figure 5B). Collectively, these results indicate that, although a core set of pathways is consistently enriched, each group exhibits additional context-specific processes consistent with variant-dependent regulatory programs.

##### Differential regulation of immune signaling pathways

**Figure 6:**
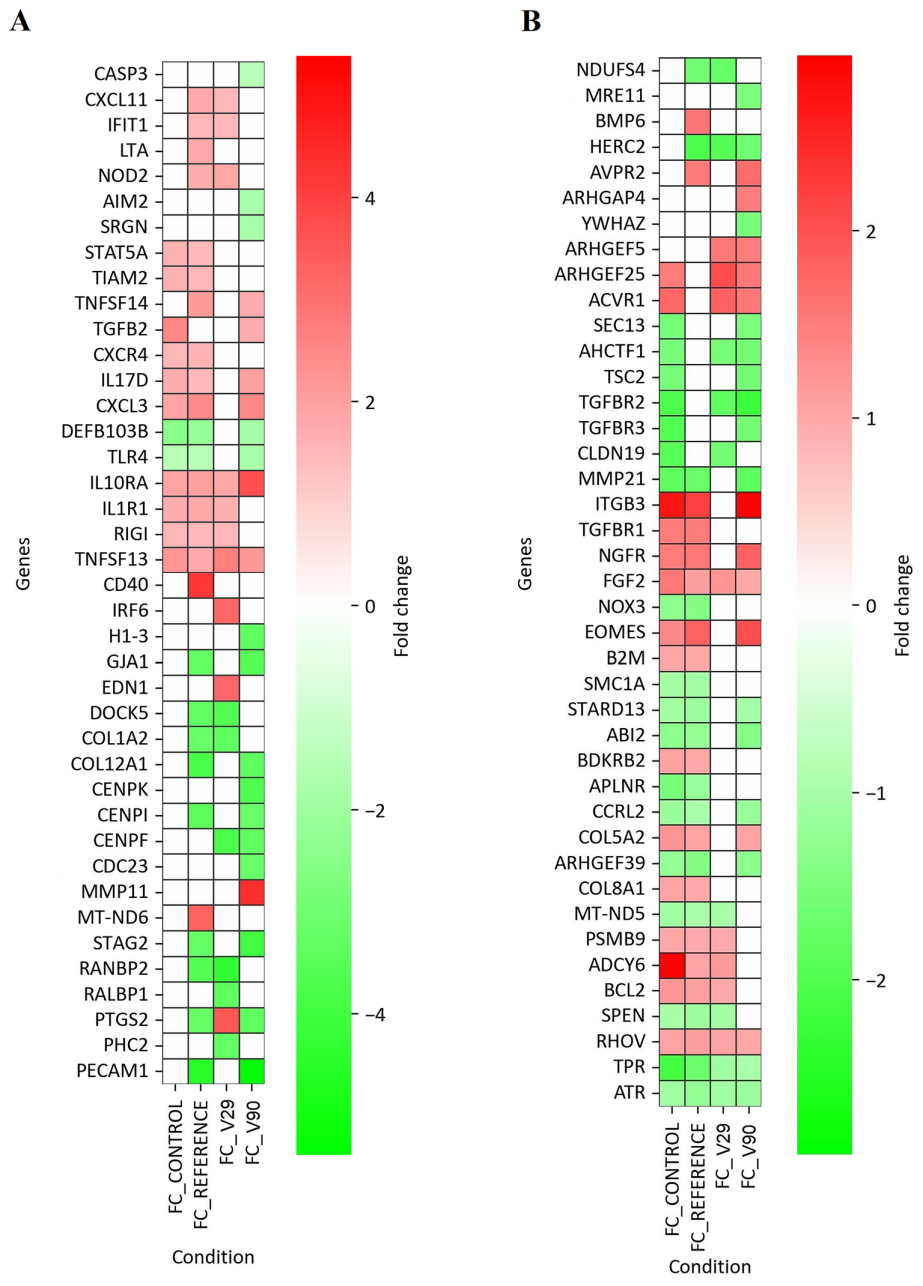
**Differential expression of immune- and cancer-related genes across study groups.** Heatmaps showing expression profiles of genes associated with immune response (**A**) and immune- and cancer-related canonical pathways (**B**). Red indicates significant upregulation, green indicates significant downregulation, and white denotes genes with no statistically significant change or no detectable expression. Differential expression was defined as fold change (FC) ≥ 1.5 or ≤ –1.5, with *p* ≤ 0.05.

To further delineate the biological impact of differentially expressed genes, subsequent analyses focused on genes mapping to immune-related canonical pathways identified across experimental groups in the context of BLV infection. Compared with the reference group, the V29 and/or V90 groups showed altered expression of genes assigned to several pathways. These included pathogen-induced cytokine storm signaling (AIM2, CASP3, CXCL11, IL17D, IL1R1, RIGI, SRGN, COL5A2, CXCL3, EOMES, LTA, NOD2, TGFB2, and TNFSF14), the role of pattern-recognition receptors in recognition of bacteria and viruses (IL17D, LTA, NOD2, RIGI, TLR4, TNFSF13, TNFSF14, and ZC3HAV1), interferon-α/β signaling (IFIT1, IRF6, IFIT3, ZC3HAV1, and IRF2BP1), the antigen presentation pathway (B2M), and macrophage classical activation signaling (CD40, CXCL11, IL17D, TLR4, TNFSF13, and TNFSF14) (Figure 6A, B). Collectively, these changes indicate that BLV miRNA variants may differentially affect core antiviral and inflammatory signaling pathways. Immune-regulatory modules showed heterogeneous regulation, including interleukin-10 signaling (IL10RA, IL1R1, and IL24), IL-17 signaling (DEFB103B, TNFSF14, IL17RA, IL17D, CXCL3, and CXCL11), granzyme A signaling (H1-3, MT-ND5, MT-ND6, and NDUFS4), and leukocyte extravasation signaling (CXCR4, NOX3, ICAM2, CXCL3, CXCL11, and CCRL2). Overall, clear differences were observed among genes involved in cytokine regulation, interferon responses, and antigen presentation. These patterns suggest that BLV miRNA sequence variants may modulate both pro- and anti-inflammatory responses, potentially influencing viral persistence and host defense.

##### Altered expression of cancer-associated and tissue remodeling pathways

In cancer-related pathways, transcripts controlling cell-cycle regulation, DNA-damage responses, and extracellular-matrix remodeling were differentially expressed, with the most substantial deviations observed in V29 and V90. Compared with the reference group, altered expression in V29 and/or V90 encompassed multiple pathways, including role of tissue factor in cancer (ACVR1, BCL2, ITGB3, TGFB2, F7, and PTGS2), SUMOylation of DNA damage response and repair proteins (HERC2, RANBP2, SMC1A, STAG2, SEC13, ATRX, FANCL, and MRE11), SUMOylation of transcription cofactors (PHC2, SPEN, STARD13, YWHAZ, KMT2A, NCOA1, NCOA2, NSD3, PIAS1, RBM15, and SETBP1), cell cycle checkpoints (AHCTF1, CENPF, CENPI, CENPK, CDC23, ATR, TPR, CDC14B, CDCA4, DNMT3A, STIL, and TSC2), molecular mechanisms of cancer (ACVR1, ADCY6, APLNR, AVPR2, BCL2, BMP6, NGFR, PTGS2, TGFBR1, TGFBR2, TGFBR3, DNMT3A, EPHA4, EPHA8, EPHB4, KIT, NSD3, and STAT5A), RHO GTPase cycle (ABI2, ARHGEF39, ARHGEF25, ARHGEF5, DOCK5, RHOV, ARHGAP4, and TIAM2), and VEGF signaling (ACVR1, ADCY6, EDN1, FGF2, ITGB3, KIT, TGFBR1, and TGFBR2) (Figure 6A, B). Together, these alterations point to variant-dependent modulation of oncogenic and cell-cycle regulatory networks, consistent with the capacity of viral miRNAs to influence tumor-associated signaling. In parallel, pathways implicated in extracellular matrix dynamics and tissue remodeling showed pronounced alterations in V29 and V90 relative to controls. Perturbed processes included assembly of collagen fibrils and other multimeric structures (COL12A1, COL8A1, COL1A2, MMP21, MMP11, COL5A2, FBLN1, FBN2, LAMA2, LAMA3, and LAMA5), extracellular matrix organization (COL12A1, COL8A1, COL1A2, MMP21, MMP11, ITGB3, PECAM1, CLDN19, GJA1, BMP6, EDN1, ADAMTS1, ADAMTS13, ADAMTS4, ADAMTS8, ADAMTSL2, CHAD, COL5A2, FBLN1, FBN2, LAMA2, LAMA3, LAMA5, RECK, and SPOCK2), collagen degradation (MMP21, MMP11, PTGS2, ADAMTS1, ADAMTS13, ADAMTS4, and ADAMTS8), and collagen biosynthesis and modifying enzymes (COL12A1, COL8A1, COL1A2, BMP6, ACVR1, TGFBR2, TGFBR3, and COL5A2). Similar changes were evident in degradation of the extracellular matrix (MMP21, MMP11, ITGB3, PTGS2, BMP6, EDN1, TGFB2, TGFBR2, TGFBR3, ADAMTS1, ADAMTS13, ADAMTS4, ADAMTS8, ADAMTSL2, RECK, and SPOCK2) and in the wound healing signaling pathway, which is frequently reprogrammed in tumors (ITGB3, PTGS2, EDN1, BMP6, ACVR1, APLNR, BDKRB2, PECAM1, GJA1, TGFBR1, TGFBR2, TGFBR3, NGFR, RALBP1, COL1A2, COL5A2, F7, MMP11, and MMP21). Taken together, these findings suggest that BLV miRNA variants may contribute to deregulation of extracellular-matrix dynamics and wound-healing programs, potentially fostering tumor-promoting microenvironmental changes.

### RT–qPCR validation of microarray data

**Figure 7:**
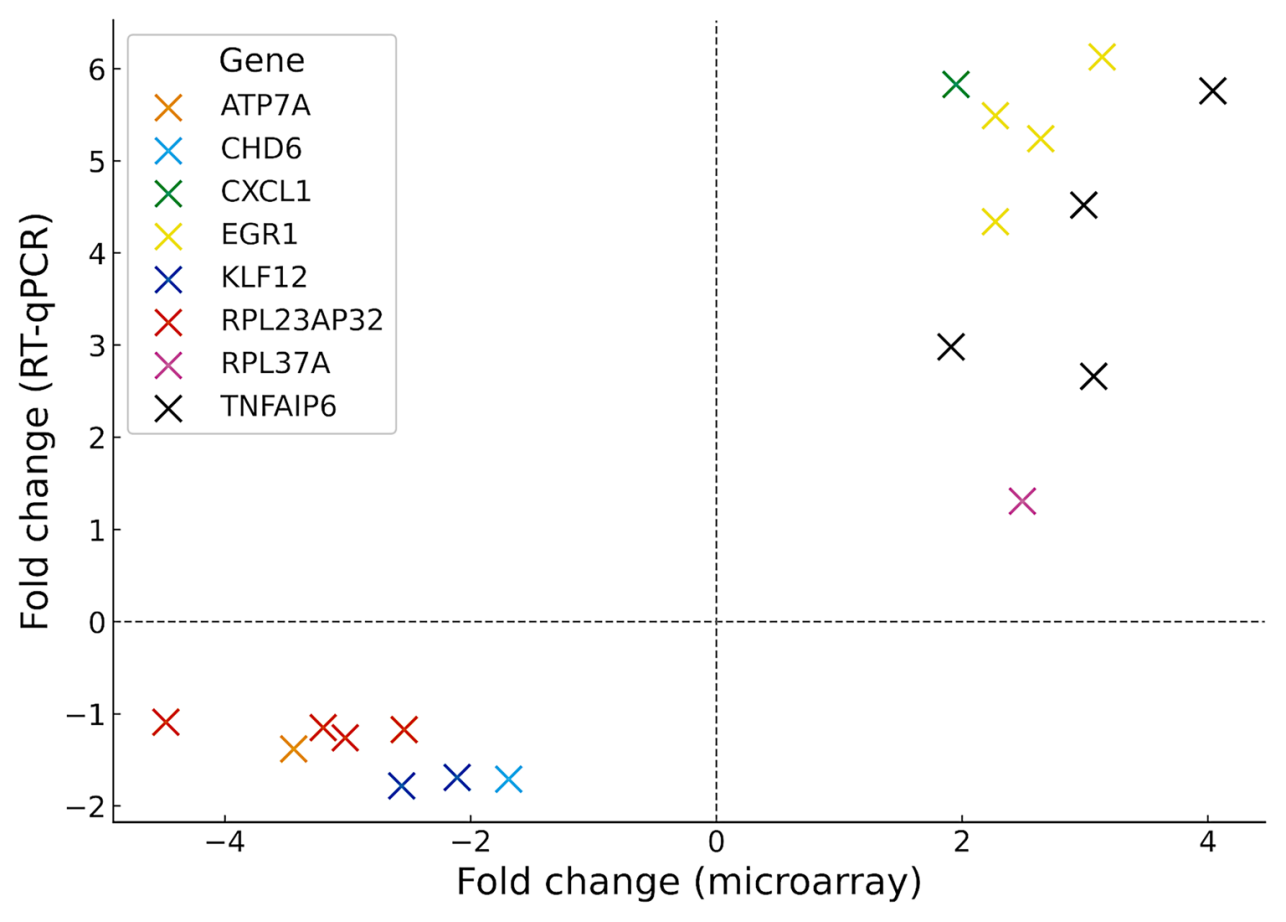
**Comparison of gene-expression fold changes measured by microarray and RT–qPCR.** Scatter plot illustrating the correlation between fold-change values obtained using both platforms across all experimental groups. Data points correspond to individual gene–group combinations.

RT–qPCR validation confirmed the expression patterns observed in the microarray data, with most genes showing consistent up- or downregulation across experimental groups (control 2, reference 344, V29, and V90) (Figure 7). RT–qPCR was performed for 18 randomly selected gene–group combinations to verify representative trends identified in the microarray analysis (Additional file 9). For example, EGR1 and TNFAIP6 were upregulated by both methods, whereas ATP7A, RPL23AP32, and KLF12 showed concordant downregulation.

### Predicted structural and accessibility differences of miRNA variants

**Figure 8:**
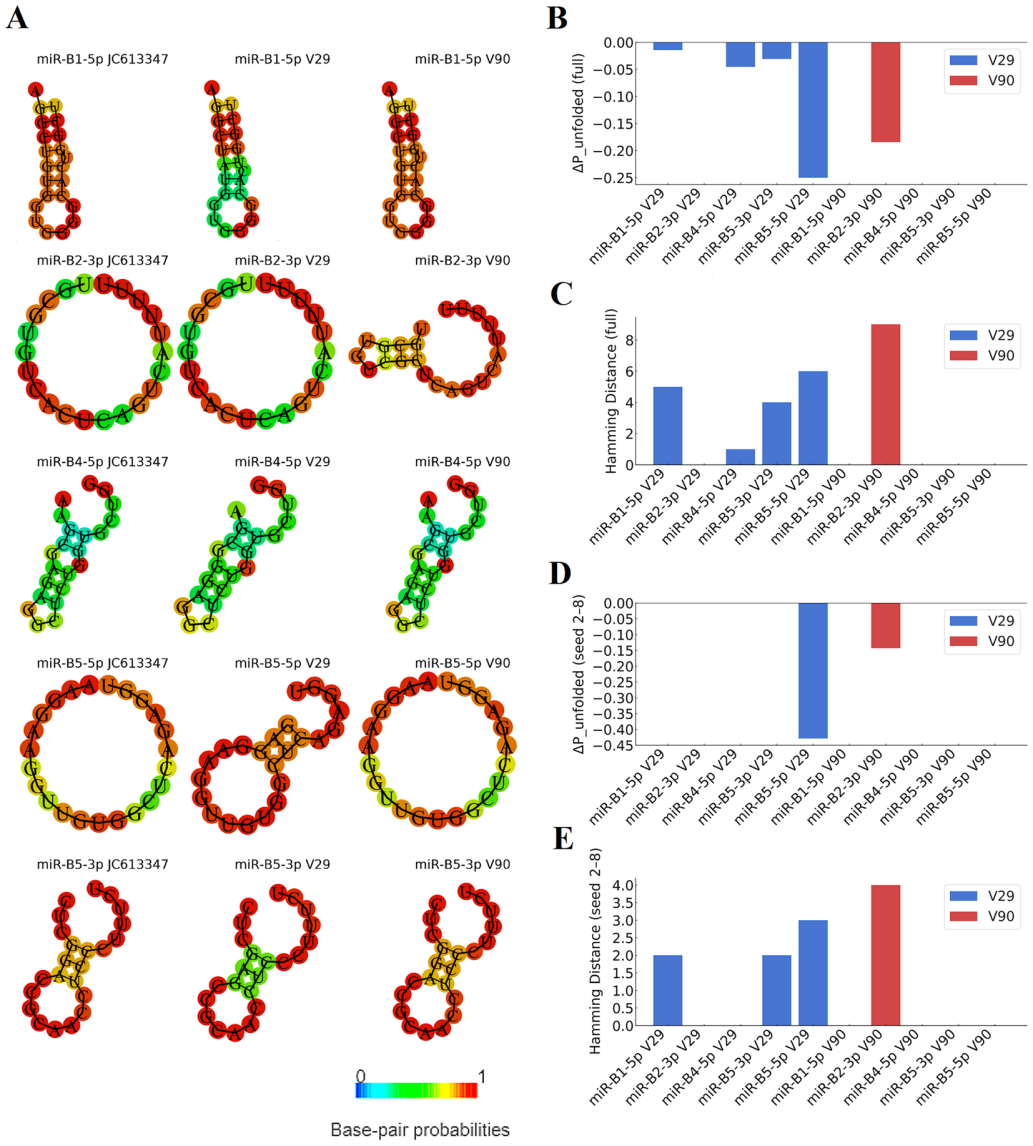
**Predicted secondary structures and comparative metrics for mature BLV miRNAs.** Predicted secondary structures for miR-B1-5p, miR-B2-3p, miR-B4-5p, miR-B5-5p, and miR-B5-3p in the reference isolate (JC613347) and variants V29 and V90. **B** Differences in structural accessibility (ΔP_unfolded) for full-length mature miRNAs. **C** Hamming distance between full-length secondary structures of each variant and the reference. **D** ΔP_unfolded restricted to the seed region (nt 2–8). **E** Hamming distance for the seed region.

Differences in host gene expression between the reference construct and variants V29 and V90 may reflect changes in the thermodynamic behavior of the corresponding mature miRNAs. Thermodynamic ensemble predictions covering minimum free-energy secondary structures, target-site accessibility, and comparative metrics are summarized in Figure 8A–E. Variant-specific SNPs within mature miRNA sequences produced detectable changes in predicted structures and accessibility, both across the full miRNA (nt 2–23) and within the seed region (nt 2–8), consistent with potential differences in target engagement and regulatory efficacy. The greatest Hamming distances, indicating the largest divergence in predicted base-pairing, were observed for miR-B2-3p (V90), miR-B5-5p (V29), and miR-B1-5p (V29), with changes also evident within seed regions. Consistently, ΔP_unfolded analyses indicated reduced structural accessibility for miR-B5-5p (V29) and miR-B2-3p (V90), suggesting diminished target-site engagement efficiency.

### Impact of seed-region polymorphisms in BLV miRNAs on host gene regulation

**Figure 9:**
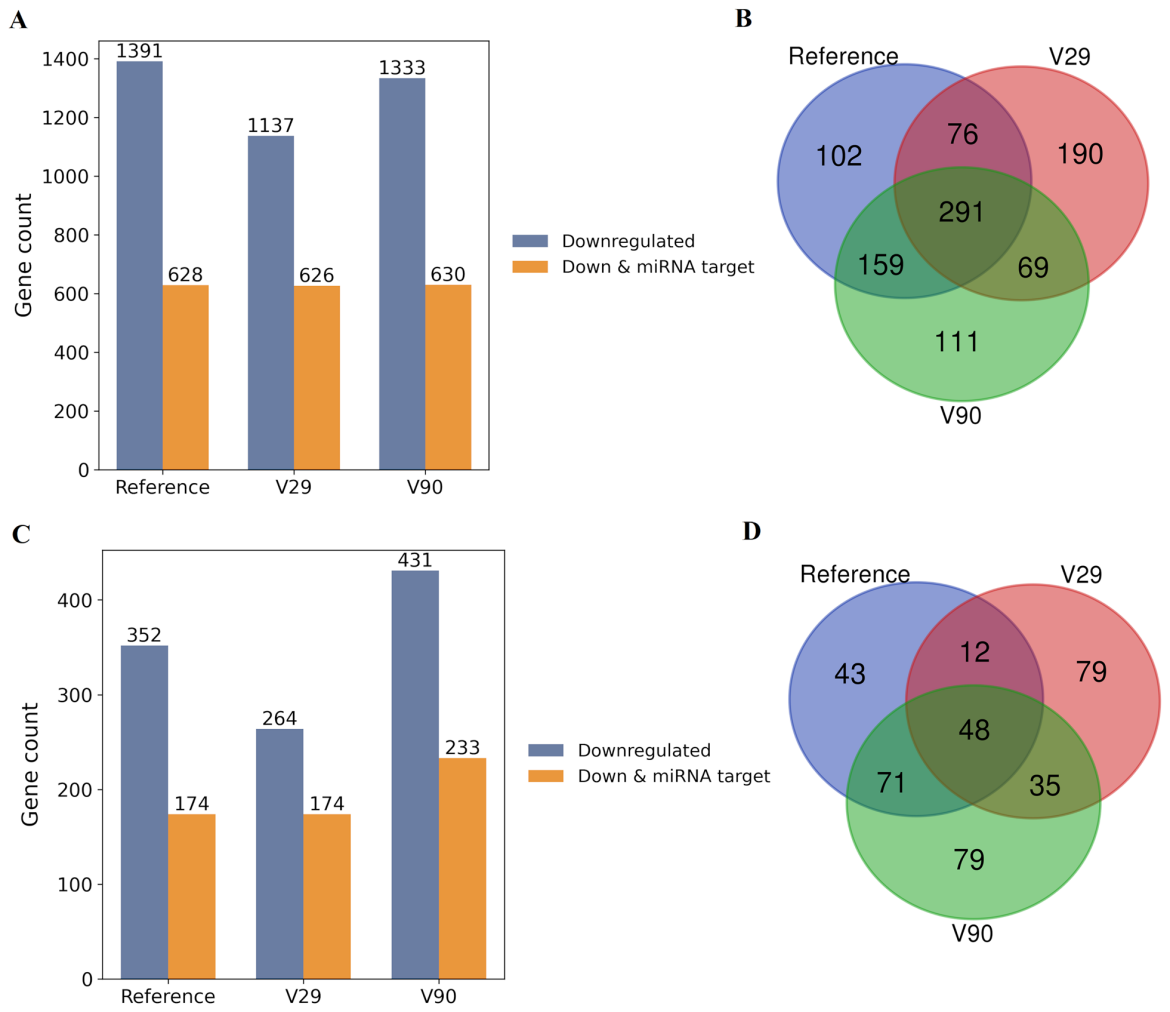
**Scope and specificity of BLV miRNA-associated host gene repression.** **A** Bar plots showing, for each group (reference, V29, and V90), the numbers of significantly downregulated genes, and the number of downregulated genes additionally predicted as BLV miRNA targets, based on comparisons with control 1 (mock). **B** Overlap of downregulated genes predicted to be BLV miRNA targets among the three groups. **C**, **D** Equivalent analyses relative to control 2.

Polymorphisms within the seed region and adjacent positions provide a direct mechanism for altering miRNA function, typically disrupting recognition of canonical targets while enabling novel interactions. In this dataset, SNPs located within the seed (nt 2–8) and immediate flanking positions (±2 nt) were detected in isolate V29 (miR-B1-5p, miR-B4-5p, miR-B4-3p, miR-B5-5p, and miR-B5-3p) and in isolate V90 (miR-B2-3p) (Table 2). To assess functional consequences, predicted targets of variant miRNA sequences, using miRanda, were intersected with genes downregulated in the microarray profiles of the reference, V29, and V90. This was carried out under the premise that direct miRNA effects are most likely reflected among repressed genes, while acknowledging that miRNAs can also act predominantly at the translational level. This integrative analysis enables prioritization of candidate miRNA–mRNA interactions rewired by seed polymorphisms. miRanda predictions indicated that approximately half of the downregulated mRNAs and lncRNAs represent putative BLV miRNAs targets, including both reference and SNP-bearing sequences. Comparable fractions of predicted targets were observed across groups: reference 45.1%, V29 55.0%, and V90 47.2% (Figure 9A, B; Additional file 10A). Hypergeometric and Fisher’s exact tests confirmed that the overlap between predicted BLV miRNA targets and downregulated genes exceeded random expectation in the reference and V29 groups, whereas V90 exhibited a weaker enrichment signal (Additional file 10B). To distinguish miRNA- associated repression from background transcriptional effects, downregulated genes in each group were compared with those observed in control 2. The majority of downregulated genes overlapped with control 2 (67.6–76.8%), consistent with cellular responses to plasmid transfection and pBLVΔ-miRNA backbone expression. However, a substantial subset of downregulated genes was specific to miRNA-expressing conditions and absent from control 2 (25.3% for reference, 23.2% for V29, and 32.4% for V90) (Figure 9C, D). Notably, miRanda predicted BLV miRNA binding sites in a large proportion of both shared and miRNA-specific genes, indicating that BLV miRNAs may reinforce repression initiated by additional mechanisms rather than acting as exclusive drivers of downregulation.

Functional enrichment analysis of downregulated predicted targets using g:Profiler revealed distinct biological signatures across groups, with developmental programs predominating in the reference set, cell-cycle and RNA-metabolism processes in V29, and immune and cell-death pathways in V90 (Additional file 11A-C). Several downregulated predicted targets corresponded to genes with established tumor-suppressive functions (Table 4). Collectively, these findings indicate that seed-region polymorphisms in BLV miRNAs are associated with variant-specific differences in the spectrum of tumor-suppressor genes among predicted downregulated targets.

**Table 4:** **Tumor-suppressor genes (TSG) among downregulated transcripts predicted to be BLV miRNA targets in the reference, V29, and V90 groups**

Group	Canonical TSG	Caretaker TSG	Context-dependent TSG
Reference	NF1, ATRX, TET2, BCOR	ATR, FANCB, SAMHD1, MTAP	CDH13, PRDM5, PTPRK, QKI, STARD13 (DLC2), MAGI1, FAT4, HIPK2, AMBRA1, DMTF1 (DMP1), SPEN, PHLPP2, SMAD2, RB1CC1 (FIP200), ZEB2
V29	PTEN, TGFBR2, NF1, ATRX, TET2, BCOR	ATR, MTAP	CDH13, PRDM5, PTPRK, QKI, STARD13 (DLC2), MAGI1, FAT4, VGLL4, AMBRA1, SPEN, PHLPP2, SMAD2, ZEB2, DMTF1 (DMP1)
V90	TSC2, TGFBR2, NF1, TET2, BCOR, PHF6	ATR, MRE11, FANCL, FANCB, SHPRH, SAMHD1, MTAP, SPIDR	CDH13, PRDM5, PTPRK, QKI, STARD13 (DLC2), MAGI1, FAT4, HIPK2, VGLL4, AMBRA1, DMTF1 (DMP1), SPEN, PHLPP2, SMAD2, ZEB2, TGFBR3, FBXO11, RASAL2, RASL10B, ARHGAP29, RAI2, ULK2, UHRF2

Genes are classified as canonical tumor suppressors, caretaker genes, or context-dependent tumor suppressors.

Several antisense lncRNAs meeting both criteria of downregulation and predicted BLV miRNA targeting, including CDKN2B-AS1, TSIX, RUNX2-AS1, KCNMA1-AS1, and GABPB1-AS1, were identified across groups, indicating that BLV miRNA variants may also modulate antisense regulatory networks.

#### Bovine orthologs of human genes

**Figure 10:**
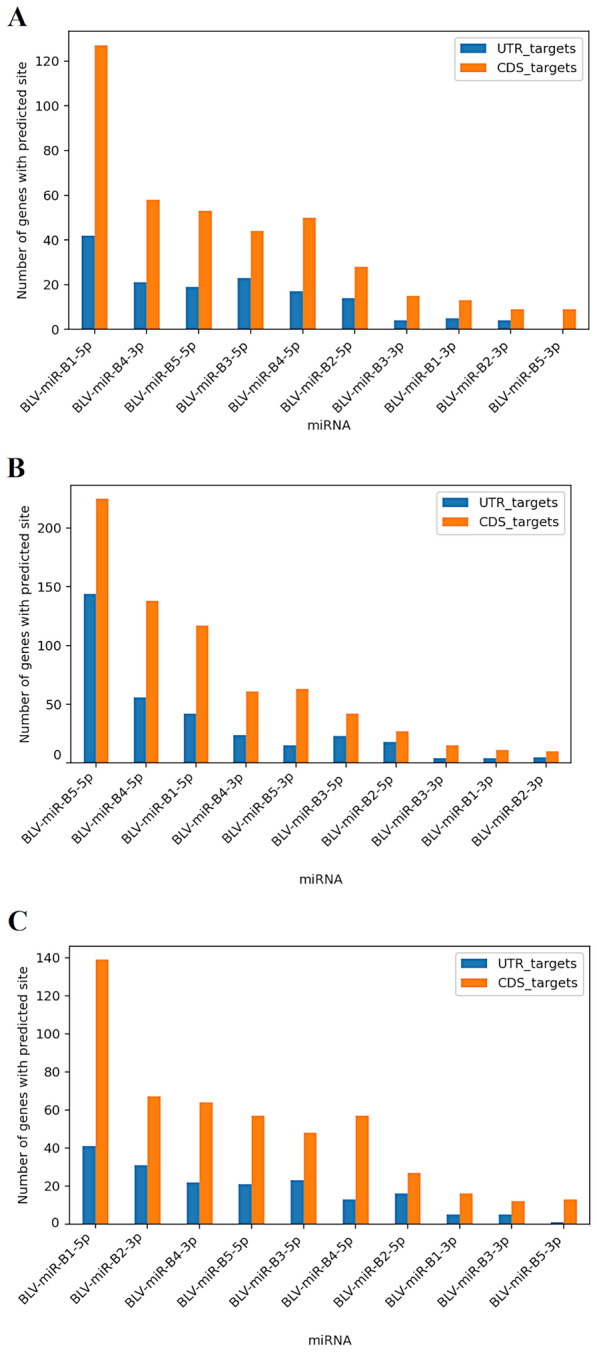
**Distribution of predicted BLV miRNA binding sites across 3′UTR and coding sequence regions in bovine orthologs.** **A** Reference BLV miRNA sequences; **B** V29 variant; **C** V90 variant.

Bovine orthologs of downregulated HEK293T genes predicted to be BLV miRNA targets were assessed for retained predicted targeting using miRanda, with 3′UTR and coding sequence regions analyzed independently within each species. Predicted binding by the same BLV miRNAs was identified for 285 (45.4%) orthologs in the reference group, 377 (60.2%) in V29, and 324 (51.4%) in V90 (Additional file 12A–C). Among bovine orthologs with predicted targeting, binding sites were distributed across both transcript regions. In the reference group, 16.5% of sites were located exclusively in the 3′UTR, 60.3% exclusively in the coding sequence, and 23.2% in both regions. Comparable distributions were observed in V29, where 12.0% of sites were located in the 3′UTR, 41.6% in the coding sequence, and 46.4% in both regions, and in V90, where the corresponding proportions were 13.9%, 58.9%, and 27.2%, respectively. The distribution of predicted binding sites across 3′UTR and coding sequence regions for individual BLV miRNAs is shown in Figure 10A–C.

## Discussion

The present study demonstrates that naturally occurring SNPs within the BLV miRNA cluster have measurable functional consequences. Sequence variation was associated with altered accumulation of selected mature miRNAs, shifts in predicted target repertoires, and remodeling of host transcriptional programs affecting both protein-coding genes and lncRNAs. These findings indicate that polymorphism within the BLV miRNA locus can modulate host gene regulation at multiple levels. A notable feature of the identified variants is the high density of substitutions and small insertions/deletions within type 2 Pol III promoter elements, particularly A- and B-box motifs. Variant V29, which harbors multiple alterations within these regulatory elements, exhibited reduced expression of several BLV miRNAs, whereas V90 showed selective attenuation of miR-B2-3p. In contrast, variants with more limited motif changes retained miR-NA levels comparable to the reference sequence. This pattern supports a functional link between Pol III promoter polymorphisms and miRNA output. BLV appears to employ an ultracompact and partially degenerate Pol III promoter architecture, characterized by overlapping and redundant A- and B-box motifs embedded within the miRNA cluster [13]. Such structural redundancy may represent an evolutionary strategy to ensure sustained miRNA transcription under strong genomic constraints, allowing abundant production of regulatory RNAs without reliance on canonical viral gene expression. In this framework, promoter motif degeneracy is not merely tolerated variation but may constitute a mechanism for fine-tuning miRNA output and, consequently, host–virus interactions.

Although secondary structure is classically discussed in the context of pre-miRNA processing, our analysis deliberately focused on mature miRNA sequences. Pre-miRNA structure primarily influences Dicer-dependent maturation efficiency, whereas regulatory specificity is ultimately determined at the level of the mature miRNA incorporated into the RISC complex [6, 27]. Sequence variation within the seed region and its immediate structural context may affect seed accessibility, Argonaute loading dynamics, and target-binding affinity [28]. Thus, analysis of mature miRNA structure complements biogenesis-focused considerations and is directly relevant to variant-dependent differences in target regulation.

Because miRNAs act at the post-transcriptional level, their regulatory effects do not uniformly translate into measurable reductions in mRNA abundance. While many targets undergo deadenylation and decay, others are regulated predominantly at the level of translation [27]. Consequently, transcriptome-based profiling captures only a subset of direct miRNA effects and inevitably includes secondary downstream changes. Comparison with control 2 allowed us to distinguish miRNA-independent transcriptional effects associated with viral backbone expression from miRNA-associated changes. Although the majority of downregulated transcripts overlapped with control 2, a consistent fraction was specific to miRNA-expressing conditions. Importantly, overlap with control 2 does not exclude additional miRNA-mediated repression, as transcriptomic classification reflects directionality rather than magnitude of change. Moreover, enrichment of predicted binding sites among downregulated genes supports a nonrandom contribution of BLV miRNAs to the observed transcriptional landscape.

The other objective of this study was to characterize cellular programs remodeled by reference versus variant miRNAs. Pathway analyses revealed a recurrent core of affected processes across experimental groups, prominently involving transcriptional regulation, rRNA processing, extracellular-matrix organization, and cytokine-related signaling. Variant-specific trends were evident. V29 was predominantly associated with cell-cycle regulation and RNA metabolic processes, whereas V90 showed stronger enrichment of immune- and inflammation-related pathways together with ECM remodeling, angiogenesis, and cancer-associated signaling modules. These patterns suggest that BLV miRNA variants may differentially bias host transcriptional programs toward proliferative or immunoregulatory states, potentially influencing the balance between viral persistence and host defense. A notable feature of the resulting landscape is the recurrent modulation of innate immune signaling components. Integration of microarray and target-prediction analyses indicates that BLV miRNAs, including seed-variant forms, are associated with altered expression and/or predicted targeting of key nodes within pathogen-recognition and interferon signaling pathways, including sensors and downstream effectors such as ZC3HAV1 (PARP13), IFIT1, IFIT3, RIG-I (DDX58), NOD2, and TLR4 [29, 30]. Modulation of B2M is consistent with potential effects on MHC class I complex stability and antigen presentation, which may influence cytotoxic T cell surveillance [31]. Similarly, changes involving IL10RA and components of the IL-17 axis (IL17D, IL17RA, CXCL3, and CXCL11) point to a reconfiguration of pro- and anti-inflammatory signaling networks that could be compatible with enhanced viral persistence [32]. This immunologic signature is consistent with strategies described for other oncogenic viruses. In EBV, BART-family miRNAs attenuate antigen recognition and presentation, supporting viral latency [33]. KSHV K12 miRNAs target components of pattern recognition and interferon signaling pathways, contributing to immune evasion and maintenance of latency [34]. Similarly, MCMV miRNAs modulate NK- and CTL-mediated recognition, prolonging survival of infected cells [35]. In HCV, interactions with host miR-122 and sequence variability within the 5′ UTR influence interferon sensitivity and promote chronic infection [36]. Although functional validation was beyond the scope of the present study, the transcriptional patterns observed here are compatible with analogous immune-modulatory strategies. We propose that SNP-associated changes in BLV miRNAs may influence both immune recognition and survival of infected cells. Variant-dependent retargeting is consistent with reduced expression and/or predicted direct targeting of components of pathogen recognition, interferon signaling, and antigen-presentation pathways, potentially diminishing the immunologic visibility of infected cells.

Beyond these methodological considerations, our findings are consistent with a model in which BLV miRNA polymorphisms preferentially shape the host environment by attenuating innate immune signaling and reducing the immunologic visibility of infected cells rather than by directly enhancing immediate viral gene expression. A cellular state characterized by diminished inflammatory and interferon responses could plausibly decrease immune-mediated clearance and thereby support longer-term proviral persistence with episodic reactivation under permissive conditions [22]. Similar miRNA-mediated modulation of host immunity to favor persistence, combined with limited but strategic reactivation, has been described for KSHV and EBV [37].

Beyond immune modulation, our data also reveal remodeling of extracellular-matrix and tissue-repair-associated pathways. Variants V29 and V90 and, to a lesser extent, the reference, were associated with altered expression of genes encoding collagens (COL1A2, COL5A2, and COL12A1), metalloproteinases (MMP11 and MMP21), integrins (ITGB3), laminins, and regulators of angiogenesis, including components of the VEGF axis, as well as mediators of tissue repair such as PTGS2 (COX-2) and EDN1. Such transcriptional patterns are compatible with a microenvironment characterized by enhanced matrix remodeling, leukocyte trafficking, and angiogenic signaling. In the context of BLV infection, these changes may contribute to conditions that favor clonal expansion of B lymphocyte populations with transformation potential [38]. It should be noted that pathways related to collagen biosynthesis and extracellular-matrix organization are not classical autonomous functions of B lymphocytes. However, matrix remodeling, stromal interactions, and angiogenic signaling are increasingly recognized as important components of the lymphoid microenvironment and leukemogenic niches. Thus, even if some of the identified transcripts are not directly linked to intrinsic B cell programs, their modulation may reflect alterations in the broader cellular context that supports B cell survival and expansion. Moreover, although BLV displays a clear tropism for B lymphocytes, viral miRNAs are highly expressed and stable, and accumulating evidence suggests that they may be released in extracellular vesicles or protein complexes [39]. Such extracellular dissemination raises the possibility that BLV miRNAs could exert paracrine effects on neighboring stromal or immune cells, thereby contributing to remodeling of the infection microenvironment.

Integration of downregulated genes with miRNA target predictions further indicates that a substantial fraction of repression is concordant with predicted BLV miRNA binding sites. Notably, several affected genes correspond to established tumor suppressors, including canonical regulators such as PTEN, TSC2, NF1, and TGFBR2, as well as genome-maintenance factors, including ATR, MRE11, FANCL, SAMHD1, and MTAP [40]. This pattern suggests that BLV miRNAs may influence networks that normally constrain proliferation and genomic stability. In addition to protein-coding genes, antisense lncRNAs such as CDKN2B-AS1 (ANRIL), TSIX, and RUNX2-AS1, were identified among downregulated predicted targets, suggesting that miRNA-mediated repression may extend beyond protein-coding genes and potentially affect lncRNA-dependent transcriptional and chromatin-associated regulatory circuits [41]. These observations align with a broader paradigm in small-RNA virology, in which viral miRNAs contribute to immune evasion while simultaneously reshaping proliferative and survival pathways [42]. In this context, the stronger enrichment patterns observed for variants V90 and V29 support a model in which naturally occurring SNPs within the BLV miRNA cluster may modulate the breadth and intensity of host–network interference, potentially influencing long-term persistence and transformation risk.

A considerable proportion of differentially expressed human transcripts possess bovine orthologs in which predicted BLV miRNA binding sites are retained. This observation suggests that at least part of the regulatory architecture observed in HEK293T cells may extend to the natural bovine host, although direct functional validation in bovine systems will be required. Nevertheless, a limitation of the present study is that transcriptome profiling was performed in the human-derived HEK293T cell line rather than in the natural bovine host cells of BLV. Species-specific differences in transcript architecture and regulatory networks may influence miRNA–target interactions, and therefore the observed transcriptional changes cannot be assumed to fully recapitulate responses in bovine B lymphocytes. However, the primary objective of this work was to perform a controlled comparative analysis of naturally occurring BLV miRNA sequence variants under standardized experimental conditions. HEK293T cells provide a highly transfectable and reproducible system that enables robust Pol III-driven miRNA expression, accurate quantification of mature miRNAs, and direct comparison of variant-dependent effects while minimizing variability associated with proviral load, infection stage, or host genetic background. Consequently, our conclusions focus on relative differences between BLV miRNA variants rather than on absolute, host-specific transcriptional outcomes. To partially address cross-species considerations, we incorporated orthology-based mapping and evaluated predicted targeting of bovine orthologs, emphasizing conserved pathways rather than individual gene effects. In the present study, miRNA target prediction analyses included both 3′UTR and coding sequence regions for human and bovine transcriptomes, analyzed independently within each species. Although 3′UTR sites are classically associated with miRNA-mediated mRNA destabilization, increasing evidence indicates that functional miRNA interactions also occur within coding sequence regions. Transcriptome-wide Argonaute binding studies and CLASH analyses have demonstrated substantial miRNA occupancy outside 3′UTRs, including within coding regions, where binding can contribute to translational repression [43]. Comparative analyses further suggest that CDS-located miRNA binding sites are subject to evolutionary constraint and are not randomly distributed. Therefore, inclusion of CDS regions in our analysis was intended to capture the full spectrum of potential miRNA–mRNA interactions rather than to inflate cross-species overlap. Notably, predicted binding sites in bovine orthologs were distributed across both transcript compartments, with a proportion located exclusively in 3′UTRs and a substantial fraction within coding sequence or spanning both regions. While 3′UTR sites may have a stronger association with transcript destabilization, CDS-localized interactions may contribute to regulatory fine-tuning. Nevertheless, functional validation will be required to determine the biological relevance of individual predicted interactions. An additional limitation concerns the transfection-based system. Differential expression of the BLV miRNAs could not be directly correlated with each polymorphic profile in vivo because high-quality RNA was not consistently retained from the corresponding PBL samples; instead, these effects were evaluated experimentally using the cloned variant loci. Although the pDrive_miRNA and pBLVΔ-miRNA constructs recapitulate key regulatory elements, expression levels under transient conditions may not fully reflect those of an integrated provirus. Comparable *tax*, *rex*, *gag*, and *env* transcript levels across variants mitigate major backbone-related effects; however, validation using replication-competent virus in bona fide target cells would strengthen physiological relevance. miRNA target predictions generated using miRanda remain inferential. Although enrichment analyses indicated that the overlap between predicted targets and downregulated transcripts exceeded random expectation, and conservative parameters beyond canonical 7-nt seed matching were applied, in silico predictions alone cannot establish direct regulation. Functional validation using 3′ UTR reporter assays, site-directed mutagenesis, or Argonaute-based CLIP approaches will be necessary to confirm specific miRNA–mRNA interactions.

Several priority directions emerge from this work. Validation in bovine B cell systems will be essential to confirm variant-dependent effects on miRNA maturation, RISC loading, and innate immune responses. Direct mapping of miRNA–mRNA interactions using Argonaute-based CLIP approaches and 3′UTR reporter assays for selected high-priority targets will clarify the extent of direct regulation. Finally, large-scale sequencing of BLV miRNA clusters in cattle cohorts, coupled with clinical phenotyping, will be required to determine epidemiologic relevance of naturally occurring SNP variants.

## Conclusions

Naturally occurring SNPs within the BLV miRNA cluster represent functionally relevant sources of regulatory diversity rather than neutral sequence variation. Our data demonstrate that selected polymorphisms can influence mature miRNA abundance and alter predicted target repertoires, and are associated with distinct host transcriptional signatures in a controlled experimental system. In particular, variants harboring seed-region changes show evidence of modified host gene targeting, including pathways linked to immune regulation, cellular stress responses, and tumor-suppressor networks. Importantly, host response to BLV infection is multifactorial and shaped by complex interactions between viral genotype, host genetics, immune status, and environmental factors. Therefore, miRNA polymorphisms should not be viewed as standalone predictors of disease outcome. Rather, they represent one component of the broader viral genetic landscape that may contribute to inter-strain variability in host–virus interactions. Further studies in bovine systems and longitudinal in vivo analyses will be required to determine the extent to which naturally occurring miRNA variants influence proviral load, immune phenotypes, or clinical progression. Within this framework, BLV miRNA polymorphisms provide a mechanistic foundation for understanding how small-scale viral sequence variation can modulate host regulatory networks and potentially shape viral persistence strategies.

## Supplementary Information

**Additional file 1. BLV isolates analyzed in this study.** This table summarizes 53 BLV-positive peripheral blood lymphocyte (PBL) samples collected from cattle in Poland between 2013 and 2019. Viral isolates were subjected to sequencing of the genomic region encoding viral microRNAs. The resulting nucleotide sequences, together with their assigned genotypes, have been deposited in GenBank under the accession numbers indicated.

**Additional file 2. Primer sequences used for qPCR amplification of BLV and human miRNAs.** Table lists the mature sequences of BLV-encoded miRNAs (blv-miR-B1–B5) and human reference miRNAs (hsa-miR-20a-5p and hsa-miR-34a-5p), together with their accession identifiers, annealing temperatures, and the forward and reverse primers used for quantitative PCR assays. All primer sequences are shown in the 5′–3′ orientation.

**Additional file 3. Primers used for RT-qPCR quantification of viral mRNA expression in HEK293T cells.**

**Additional file 4.** **List of primers used in RT-qPCR assays.**

**Additional file 5.** **Alignment of BLV sequences with annotated regulatory elements and nucleotide variants.** The alignment of 53 BLV sequences relative to the reference sequence JC613347 is presented. Sequence reads V1–V53 display nucleotide variants in comparison to the reference. Colored rectangles positioned above the sequences indicate the locations of specific regulatory elements, including the A-box-like and A*-box-like, shown in dark green and light green respectively, the B-box-like and B*-box-like, shown in blue, the Seed regions, highlighted in orange, and the Termination signal sequences, marked in red. In addition, auxiliary elements BLV-mir-B1–B5 and B1–B5 pre-miRNA are indicated in dark gray and light gray, respectively. Colored letters within the sequences denote nucleotides that differ from the reference sequence, with the applied color scheme corresponding to the type of nucleotide substitution.

**Additional file 6.** **Localization and characterization of nucleotide polymorphisms within regulatory elements located in the miRNA loci of the BLV sequence.**

**Additional file 7.** **Comprehensive list of microarray-identified differentially expressed genes across Control 2, Reference, V29 and V90 groups with identifiers, regulation status and fold-change metrics.**

**Additional file 8.** **Group-specific differentially expressed genes identified by microarray analysis.** This file contains four worksheets (A–D) presenting transcripts uniquely expressed in the Control 2, Reference, V29, and V90 groups, respectively. Each table includes gene symbols, accession numbers, fold-change values, and functional annotations. The data summarize group-specific gene expression profiles and highlight key biological processes, including extracellular matrix organization, immune and inflammatory responses, metabolic regulation, cytoskeleton dynamics, and genome maintenance.

**Additional file 9.** **Expression analysis of selected genes in different groups—microarray and RT-qPCR results.**

**Additional file 10. Predicted miRNA target genes and enrichment analysis**. This file contains two worksheets (A–B). (A) presents host genes predicted to be directly targeted by BLV-encoded miRNAs and the proportion of repressed genes attributable to direct miRNA targeting in each isolate (Reference, V29, V90). (B) shows enrichment analysis of miRNA target genes within differentially expressed genes using hypergeometric and Fisher’s exact tests.

**Additional file 11.** **Functional enrichment analysis (g:Profiler) of host genes predicted to be directly targeted by BLV miRNAs.** This file contains three worksheets (A–C) corresponding to the Reference, V29, and V90 isolates. The analysis identifies significantly enriched biological categories across Gene Ontology Biological Process (GO:BP), Cellular Component (GO:CC), and Molecular Function (GO:MF), as well as Human Phenotype Ontology (HP), Human Protein Atlas (HPA), miRNA regulatory networks (MIRNA), and WikiPathways (WP). For each enriched term, the table reports its source (annotation database), term_id (stable identifier), term_name (descriptive label), adjusted_p_value (significance after multiple testing correction), intersection_size (number of BLV miRNA target genes overlapping with that term), and intersections (specific overlapping genes). (A) shows results for the Reference isolate, in which 45.1% of repressed host genes are predicted direct BLV miRNA targets. (B) shows results for the V29 isolate, in which 55.0% of repressed host genes are predicted direct BLV miRNA targets. (C) shows results for the V90 isolate, in which 47.2% of repressed host genes are predicted direct BLV miRNA targets.

**Additional file 12**. **Bovine orthologs of human differentially expressed genes predicted as BLV miRNA targets.** This file contains three worksheets (A–C) corresponding to the Reference, V29, and V90 groups. (A) presents bovine orthologs of human differentially expressed genes in the Reference group predicted as BLV miRNA targets. (B) presents bovine orthologs of human differentially expressed genes in the V29 group predicted as BLV miRNA targets. (C) presents bovine orthologs of human differentially expressed genes in the V90 group predicted as BLV miRNA targets.

## Data Availability

The BLV miRNA locus sequences generated and analyzed in this study have been deposited in GenBank under accession nos. PV185290–PV185338 and MW470848–MW470851. The microarray dataset generated during the current study has been deposited in the NCBI Gene Expression Omnibus (GEO) under accession no. GSE299212.
