# Supplementary material for: Natural polymorphisms in the bovine leukemia virus microRNA cluster modulate miRNA expression and host regulatory pathways
Source: Vet Res. 2026 May 21;57:81. doi: 10.1186/s13567-026-01776-0 (PMC13192155; doi:10.1186/s13567-026-01776-0)
Supplement: Supplementary file 1 — Additional file 1. BLV isolates analyzed in this study. This table summarizes 53 BLV-positive peripheral blood lymphocyte (PBL) samples collected from cattle in Poland between 2013 and 2019. Viral isolates were subjected to sequencing of the genomic region encoding viral microRNAs. The resulting nucleotide sequences, together with their assigned genotypes, have been deposited in GenBank under the accession numbers indicated. [file 13567_2026_1776_MOESM1_ESM.docx]

**Additional file 1.** BLV isolates analyzed in this study. This table summarizes 53 BLV-positive peripheral blood lymphocyte (PBL) samples collected from cattle in Poland between 2013 and 2019. Viral isolates were subjected to sequencing of the genomic region encoding viral microRNAs. The resulting nucleotide sequences, together with their assigned genotypes, have been deposited in GenBank under the accession numbers indicated.

| Sequence name | Isolate name | Collection date | Genotype | GenBank  accession number | Provirus copies per 1 000 cells |
| --- | --- | --- | --- | --- | --- |
| V90 | 16Bmut107 | 2018 | G4 | PV185290 | 94.61 |
| V91 | 15B | 2018 | G4 | PV185291 | 0.32 |
| V92 | 15C | 2014 | G4 | PV185292 | 3.38 |
| V93 | 20C | 2018 | G4 | PV185293 | 4.75 |
| V94 | 12C | 2013 | G4 | PV185294 | 498.67 |
| V95 | 10C | 2017 | G4 | PV185295 | 0.17 |
| V96 | 2_mir | 2019 | G4 | PV185296 | 2.83 |
| V97 | 20A | 2017 | G4 | PV185297 | 41.50 |
| V98 | 16C | 2016 | G4 | PV185298 | 0.03 |
| V99 | 8_mir | 2019 | G4 | PV185299 | 0.43 |
| V00 | 10B | 2017 | G4 | PV185300 | 17.38 |
| V01 | 8B | 2018 | G4 | PV185301 | 2.61 |
| V02 | 17A | 2017 | G4 | PV185302 | 0.12 |
| V03 | 4B | 2018 | G4 | PV185303 | 2.71 |
| V04 | 14B | 2018 | G4 | PV185304 | 0.06 |
| V05 | 13B | 2018 | G4 | PV185305 | 0.13 |
| V06 | 4_mir | 2019 | G4 | PV185306 | 3.26 |
| V07 | 3C | 2015 | G4 | PV185307 | 186.30 |
| V08 | 13_mir | 2019 | G4 | PV185308 | 0.22 |
| V09 | 5B | 2018 | G4 | PV185309 | 0.48 |
| V10 | 11B | 2017 | G4 | PV185310 | 0.36 |
| V11 | 3_mir | 2019 | G4 | PV185311 | 0.05 |
| V12 | 11C | 2015 | G4 | PV185312 | 23.44 |
| V13 | 19B | 2013 | G4 | PV185313 | 17.45 |
| V14 | 4C | 2015 | G4 | PV185314 | 3.98 |
| V15 | 14_mir | 2019 | G4 | PV185315 | 0.37 |
| V16 | 6_mir | 2019 | G4 | PV185316 | 0.21 |
| V17 | 14C | 2013 | G4 | PV185317 | 12.29 |
| V18 | mir3 | 2019 | G4 | PV185318 | 0.23 |
| V19 | 20B | 2014 | G4 | PV185319 | 0.02 |
| V20 | 1_mir | 2019 | G4 | PV185320 | 4.26 |
| V21 | 3B | 2018 | G4 | PV185321 | 17.43 |
| V22 | 19C | 2015 | G4 | PV185322 | 216.36 |
| V23 | 1C | 2013 | G4 | PV185323 | 3.37 |
| V24 | 8C | 2016 | G4 | PV185324 | 54.33 |
| V25 | 18_mir | 2019 | G4 | PV185325 | 0.21 |
| V26 | 11_mir | 2019 | G4 | PV185326 | 0.39 |
| V27 | 9C | 2013 | G4 | PV185327 | 54.33 |
| V28 | 19_mir | 2019 | G4 | PV185328 | 0.49 |
| V29 | 3Amut74 | 2013 | G8 | PV185329 | 0.37 |
| V30 | 10A | 2016 | G7 | PV185330 | 0.06 |
| V31 | 7B | 2018 | G7 | PV185331 | 0.37 |
| V32 | 6B | 2017 | G7 | PV185332 | 0.07 |
| V33 | 1A | 2013 | G7 | PV185333 | 700.74 |
| V34 | mir4 | 2019 | G8 | PV185334 | 0.17 |
| V35 | 6A | 2015 | G8 | PV185335 | 78.13 |
| V36 | 14A | 2018 | G8 | PV185336 | 0.28 |
| V37 | 12A | 2018 | G8 | PV185337 | 0.18 |
| V38 | 9A | 2013 | G8 | PV185338 | 139.58 |
| V48 | Pol_1 | 2017 | G7 | MW470848 | 0.48 |
| V49 | Pol_2 | 2017 | G7 | MW470849 | 0.82 |
| V50 | Pol_3 | 2017 | G8 | MW470850 | 0.85 |
| V51 | Pol_4 | 2017 | G8 | MW470851 | 0.49 |

The number of provirus copies per 1 000 cells was calculated as follows: (copy number of BLV pol)/(copy number of H3F3A/2) × 1 000 cells.
