# Supplementary material for: Natural polymorphisms in the bovine leukemia virus microRNA cluster modulate miRNA expression and host regulatory pathways
Source: Vet Res. 2026 May 21;57:81. doi: 10.1186/s13567-026-01776-0 (PMC13192155; doi:10.1186/s13567-026-01776-0)
Supplement: Supplementary file 2 — Additional file 2. Primer sequences used for qPCR amplification of BLV and human miRNAs. Table lists the mature sequences of BLV-encoded miRNAs (blv-miR-B1–B5) and human reference miRNAs (hsa-miR-20a-5p and hsa-miR-34a-5p), together with their accession identifiers, annealing temperatures, and the forward and reverse primers used for quantitative PCR assays. All primer sequences are shown in the 5′–3′ orientation. [file 13567_2026_1776_MOESM2_ESM.docx]

**Additional file 2.** Primer sequences used for qPCR amplification of BLV and human miRNAs. Table lists the mature sequences of BLV-encoded miRNAs (blv-miR-B1–B5) and human reference miRNAs (hsa-miR-20a-5p and hsa-miR-34a-5p), together with their accession identifiers, annealing temperatures, and the forward and reverse primers used for quantitative PCR assays. All primer sequences are shown in the 5′–3′ orientation.

| **miRNA** | **Mature sequence (5′–3′)** | **Accession** | **Annealing T (°C)** | **Forward primer (5′–3′)** | **Reverse primer (5′–3′)** |
| --- | --- | --- | --- | --- | --- |
| blv-miR-B1-5p | AGGCTGTGGTGGTGCACTGGCTT | MIMAT0027346 | 60 | CTGTGGTGGGGCACT | GGTCCAGTTTTTTTTTTTTTTTAGC |
| blv-miR-B1-3p | TCAGTGTACCATCACAAGCCTCT | MIMAT0025859 | 60 | GTCAGTGTACCATCACAAGC | AGGTCCAGTTTTTTTTTTTTTTTAGAG |
| blv-miR-B2-5p | ATGACTGAGTGTAGCGCAGAGA | MIMAT0025860 | 60 | CGCAGATGACTGAGTGTAG | GGTCCAGTTTTTTTTTTTTTTTCTCT |
| blv-miR-B2-3p | TGCGTGTCGCTCAGTCATTTT | MIMAT0025861 | 60 | TGCGTGTCACTCAGTCA | AGGTCCAGTTTTTTTTTTTTTTTAAAATG |
| blv-miR-B3-5p | ATCCCCCTGCCAGCGTTGGTC | MIMAT0025862 | 60 | CCCCTGCCAGCGT | GGTCCAGTTTTTTTTTTTTTTTAGAC |
| blv-miR-B3-3p | TAACGCTGACGGGGGCGATTTCT | MIMAT0025863 | 60 | CTGACGGGGGCGAT | GGTCCAGTTTTTTTTTTTTTTTAGAAATC |
| blv-miR-B4-5p | GCGGGAGGCTCTGGTGCTGG | MIMAT0027347 | 60 | GCGAGAGGCTCTGGTG | GGTCCAGTTTTTTTTTTTTTTTCCA |
| blv-miR-B4-3p | TAGCACCACAGTCTCTGCGCCTTT | MIMAT0025864 | 60 | GTAGCACCACAGTCTCTG | GGTCCAGTTTTTTTTTTTTTTAAAGG |
| blv-miR-B5-5p | AGGAAGGTTGTGGCTCAGAGGT | MIMAT0025865 | 60 | GGAAGGTTGTGGCTCAG | CCAGTTTTTTTTTTTTTTTACCTCTG |
| blv-miR-B5-3p | CTCGAGCCGCAACCTCCCTTTCT | MIMAT0025866 | 60 | CTCGGACCGCAACCT | GTCCAGTTTTTTTTTTTTTTTAGAAAGG |
| hsa-miR-20a-5p | TAAAGTGCTTATAGTGCAGGTAG | MIMAT0000075 | 60 | GCAGTAAAGTGCTTATAGTGCAG | GTCCAGTTTTTTTTTTTTTTTCTACCT |
| hsa-miR-34a-5p | TGGCAGTGTCTTAGCTGGTTGT | MIMAT0000255 | 60 | GTGGCAGTGTCTTAGCTG | TCCAGTTTTTTTTTTTTTTTACAACCA |
