# Supplementary material for: Natural polymorphisms in the bovine leukemia virus microRNA cluster modulate miRNA expression and host regulatory pathways
Source: Vet Res. 2026 May 21;57:81. doi: 10.1186/s13567-026-01776-0 (PMC13192155; doi:10.1186/s13567-026-01776-0)
Supplement: Supplementary file 3 — Additional file 3. Primers used for RT-qPCR quantification of viral mRNA expression in HEK293T cells. [file 13567_2026_1776_MOESM3_ESM.docx]

**Additional file 3.** Primers used for RT-qPCR quantification of viral mRNA expression in HEK293T cells.

| Target gene | Forward primer (5’→3’) | Reverse primer (5’→3’) |
| --- | --- | --- |
| **HPRT** | GGTCAAGAAGCATAAACCAAAG | AAGGGCATATCCCACAACAAAC |
| **Tax/Rex** | GCGTTTGCTGAAAGCCTTCAA | GGGCAGGCATGTAGAGAGTG |
| **Gag** | TCCCTTTCTCATCACGTTCC | GTGGGGGTGAATGGTGTAAC |
| **Env** | CTATCCGGCAGCGGTCAG | GAGGAGAGTAAGAGTGAGACTTACCC |

Primer sequences were obtained from Gillet NA, Hamaidia M, de Brogniez A, Gutiérrez G, Renotte N, et al. (2016). **Bovine leukemia virus small noncoding RNAs are functional elements that regulate replication and crontribute to oncogenesis in vivo*.*** PLoS Pathogens, 12(4): e1005588. <https://doi.org/10.1371/journal.ppat.1005588>
