# Supplementary material for: Natural polymorphisms in the bovine leukemia virus microRNA cluster modulate miRNA expression and host regulatory pathways
Source: Vet Res. 2026 May 21;57:81. doi: 10.1186/s13567-026-01776-0 (PMC13192155; doi:10.1186/s13567-026-01776-0)
Supplement: Supplementary file 4 — Additional file 4. List of primers used in RT-qPCR assays. [file 13567_2026_1776_MOESM4_ESM.docx]

**Additional file 4.** List of primers used in RT-qPCR assays.

| Gene | Reference sequence ID | Primer sequence (5′–3′) |
| --- | --- | --- |
| HPRT1* | ENST00000298556.8 | F: GCAGACTTTGCTTTCCTTGG  R: ACACTTCGTGGGGTCCTTTT |
| ATP7A | NM_000052.7 | F: CGTAGCTTGTGATATACCAG  R: AGCCATCACACCAAGCAGAT |
| CHD6 | NM_032221 | F: CTCACCACTCTTGATCAGGT  R: TCTGAGGTCCCATCTGTAAC |
| CXCL1 | NM_001511.4 | F: TGAACTGCGCTGCCAGTGCTT  R: TGACTTCGGTTTGGGCGCAGT |
| EGR1 | NM_001964 | F: AGCCCTACGAGCACCTGAC  R: GTCTCCACCAGCACCTTCTC |
| KLF12 | NM_001400136.1 | F: AATGCTTGATGGGATGCCGG  R: AACAGGGGAACGGCTTCCAT |
| RPL23AP32 | NR_002229 | F: GTGACTGATGCAGAGTACTG  R: CTTTGAGGTAACTCTACTGGG |
| RPL37A | ENST00000446558 | F: ACCAAGATGAAGAGACGAGC  R: TTCCATTCTGTTGTACGTCC |
| TNFAIP6 | NM_007115 | F: GTGGAGATGAGCTTCCAGATG  R: GGAAACCTCCAGCTGTCACT |

*HPRT1 was used as an internal reference gene.*
