# Supplementary figures and images for: Natural polymorphisms in the bovine leukemia virus microRNA cluster modulate miRNA expression and host regulatory pathways
Source: Vet Res. 2026 May 21;57:81. doi: 10.1186/s13567-026-01776-0 (PMC13192155; doi:10.1186/s13567-026-01776-0)

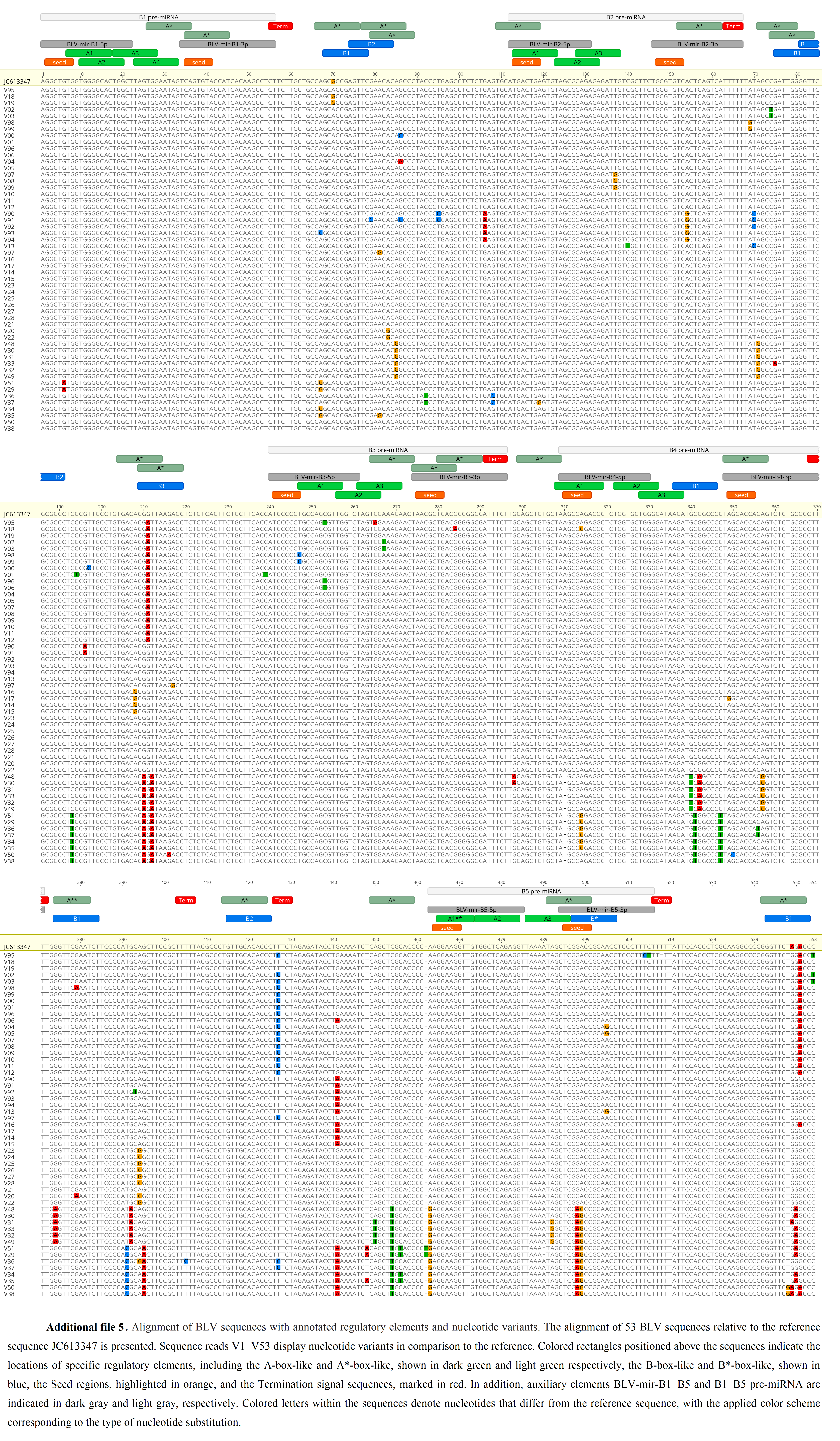

Supplement: Supplementary file 5 — Additional file 5. Alignment of BLV sequences with annotated regulatory elements and nucleotide variants. The alignment of 53 BLV sequences relative to the reference sequence JC613347 is presented. Sequence reads V1–V53 display nucleotide variants in comparison to the reference. Colored rectangles positioned above the sequences indicate the locations of specific regulatory elements, including the A-box-like and A*-box-like, shown in dark green and light green respectively, the B-box-like and B*-box-like, shown in blue, the Seed regions, highlighted in orange, and the Termination signal sequences, marked in red. In addition, auxiliary elements BLV-mir-B1–B5 and B1–B5 pre-miRNA are indicated in dark gray and light gray, respectively. Colored letters within the sequences denote nucleotides that differ from the reference sequence, with the applied color scheme corresponding to the type of nucleotide substitution. [file 13567_2026_1776_MOESM5_ESM.png]
