# Supplementary material for: Natural polymorphisms in the bovine leukemia virus microRNA cluster modulate miRNA expression and host regulatory pathways
Source: Vet Res. 2026 May 21;57:81. doi: 10.1186/s13567-026-01776-0 (PMC13192155; doi:10.1186/s13567-026-01776-0)
Supplement: Supplementary file 6 — Additional file 6. Localization and characterization of nucleotide polymorphisms within regulatory elements located in the miRNA loci of the BLV sequence. [file 13567_2026_1776_MOESM6_ESM.docx]

**Additional file 6.** Localization and characterization of nucleotide polymorphisms within regulatory elements located in the miRNA loci of the BLV sequence.

| Name | Localization | Single Nucleotide Polymorphisms (SNPs) |
| --- | --- | --- |
| BLV-mir-B1-5p | 1-22 | 6: {A: 2, G: 51} |
| Seed | 2-8 | 6: {A: 2, G: 51} |
| A*-box-like | 66-76 | 67: {A:48, G:4, C:1} |
| B1-box–like | 68-78 | 70: {A: 51, G: 2} |
| B2-box–like | 74-84 | 79: {C: 1, G: 52}; 81: {A: 51, G: 2}; 83: {A: 51, G: 2} |
| A*-box-like | 77-87 | 85: {A:47, G:6}; 86: {G: 50, C:2, A:1} |
| A*-box-like | 79-89 | 85: {A:47, G:6}; 86: {G: 50, C:2, A:1} |
| A*-box-like | 109-119 | 119: {A: 52, G: 1} |
| BLV-mir-B2-5p | 112-131 | 119: {A: 52, G: 1} |
| Seed | 113-119 | 119: {A: 52, G: 1} |
| A1-box–like | 113-123 | 119: {A: 52, G: 1} |
| A3-box–like | 128-138 | 137: {G: 3, T: 50} |
| BLV-mir-B2-3p | 146-167 | 154: {A: 48, G: 5} |
| Adjacent to Seed 147-153 | | 154: {A: 48, G: 5} |
| A*-box-like | 152-162 | 154: {A: 48, G: 5} |
| Adjacent to A*-box-like 171-180 | | 170: {T:49, C:4} |
| A*-box-like | 171-180 | 171: {A:47, G:6}; 174: {C:51, T:2} |
| A*-box-like | 174-184 | 174: {C:51, T:2} |
| B1-box–like | 175-185 | 175: {A: 1, G: 52} |
| In vicinity of B2-box–like 181-191 | | 193: {C:45, T:8} |
| A*-box-like | 204-214 | 208: {A:49, G:4} |
| B3-box–like | 209-219 | 210: {A: 14, G: 39}; 211: {A: 19, G: 34}; 212: {A: 14, T: 39}; 216: {A: 1, G: 52}; 217: {A: 52, G: 1} |
| A*-box-like | 209-219 | 210: {A: 14, G: 39}; 211: {A: 19, G: 34}; 212: {A: 14, T: 39}; 216: {A: 1, G: 52}; 217: {A: 52, G: 1} |
| In the vicinity of Seed 241-247 | | 239: {C:52, T:1} |
| BLV-mir-B3-5p | 240-261 | 247: {C: 2, T: 51}; 253: {C: 50, T: 3} |
| Seed | 241-247 | 247: {C: 2, T: 51} |
| A1-box–like | 247-257 | 247: {C: 2, T: 51}; 253: {C: 50, T: 3} |
| A2-box–like | 256-266 | 265: {A: 1, G: 52} |
| A3-box–like | 261-271 | 265: {A: 1, G: 52}; 267: {A: 51, T: 2} |
| A*-box-like | 264-274 | 265: {A: 1, G: 52}; 267: {A: 51, T: 2} |
| BLV-mir-B3-3p | 274-296 | 284: {A: 1, G: 52} |
| A*-box-like | 274-284 | 284: {A: 1, G: 52} |
| A*-box-like | 280-290 | 284: {A: 1, G: 52} |
| Adjacent to A*-box-like 299-309 | | 298: {G:51, A:2} |
| Adjacent to A*-box-like 299-309 | | 310: {del: 14, A: 39} |
| BLV-mir-B4-5p | 309-330 | 310: {del: 14, A: 39}; 314: {A: 46, G: 7} |
| Seed | 310-316 | 310: {del: 14, A: 39}; 314: {A: 46, G: 7} |
| A1-box–like | 308-319 | 310: {del: 14, A: 39}; 314: {A: 46, G: 7} |
| B1-box–like | 336-346 | 340: {G: 47, T: 6}; 341: {C: 45, T: 8}; 342: {A: 6, G: 47} |
| BLV-mir-B4-3p | 348-371 | 350: {A: 52, G: 1}; 356: {C: 51, T: 2}; 357: {A: 47, G: 6} |
| In the vicinity of A*-box-like 348-358 and Seed 349-355 | | 347: {C: 47, T:8} |
| A*-box-like | 348-358 | 350: {A: 52, G: 1}; 356: {C: 51, T: 2}; 357: {A: 47, G: 6} |
| Seed | 349-355 | 350: {A: 52, G: 1}; |
| B1-box–like | 374-384 | 374: {A: 6, G: 47}; 379: {G:51, A:2} |
| A**-box-like | 374-382 | 374: {A: 6, G: 47}; 379: {G:51, A:2} |
| Termination | 403-407 | 405: {C: 1, T: 52} |
| Termination | 426-430 | 427: {C: 20, T: 33} |
| Adjacent to A*-box-like 449-459 | | 448: {T:50, A:3} |
| A*-box-like | 449-459 | 450: {A:49, T:4}; 454: {C:39, T:14}; 456: {C:49, T:4} |
| BLV-mir-B5-5p | 463-485 | 463: {A: 39, G: 14} |
| Adjacent to BLV-mir-B5-5p and Seed 464-470 | | 462: {-: 51, ins T: 2} |
| Seed | 464-470 | 463: {A: 39, G: 14} |
| A3-box–like | 486-496 | 490: {del: 2, A: 51}; 492: {A: 49, G: 4} |
| A*-box-like | 491-501 | 490: {del: 2, A: 51}; 492: {A: 49, G: 4}; 498: {A: 14, G: 39}; 499: {A: 39, G: 14} |
| BLV-mir-B5-3p | 494-516 | 498: {A: 14, G: 39}; 499: {A: 39, G: 14}; 505: {A: 50, G: 3}; 514: {C: 1, T: 52}; 515: {C: 52, T: 1} |
| Seed | 495-501 | 498: {A: 14, G: 39}; 499: {A: 39, G: 14} |
| B*-box–like | 497-507 | 498: {A: 14, G: 39}; 499: {A: 39, G: 14}; 505: {A: 50, G: 3} |
| Termination | 516-520 | 515: {C: 52, T: 1}; 518: {del: 1, T: 52} |
| B1-box–like | 543-553 | 548: {G: 2, T: 51}; 549: {A: 3, G: 50}; 550: {A: 9, G: 44}; 551: {A: 22, G: 31} |
| Adjacent to B1-box–like 543-553 | | 554: {C:50, T:3} |

The table presents a summary of single nucleotide polymorphism (SNP) positions identified within the BLV miRNA-coding region, both inside and in the vicinity of major regulatory elements, such as the A-box-like, cryptic A*-box-like, B-box-like, cryptic B*-box-like, Seed regions, Termination signal sequences, as well as within the microRNAs (BLV-mir-B1–B5). The SNPs were identified across 53 analyzed sequences in comparison to the reference sequence JC613347. For each element, the localization, sequence position range, and a detailed characterization of nucleotide variants are provided, including their type and frequency of occurrence in the analyzed sequences (formatted as ‘position: {nucleotide: count, …}’). Ins – insertion, del – deletion.
